# The CRL3^KCTD10^ ubiquitin ligase–USP18 axis coordinately regulates cystine uptake and ferroptosis by modulating SLC7A11

**DOI:** 10.1073/pnas.2320655121

**Published:** 2024-07-03

**Authors:** Qiyin Zhou, Hongfei Yu, Yongxia Chen, Jiayi Ren, Yan Lu, Yi Sun

**Affiliations:** ^a^Cancer Institute (Key Laboratory of Cancer Prevention and Intervention, China National Ministry of Education, Key Laboratory of Molecular Biology in Medical Sciences), The Second Affiliated Hospital, Zhejiang University School of Medicine, Hangzhou, Zhejiang Province 310009, China; ^b^Institute of Translational Medicine, Zhejiang University School of Medicine, Hangzhou 310029, China; ^c^Cancer Center, Zhejiang University, Hangzhou 310058, China; ^d^Zhejiang Provincial Clinical Research Center for Cancer, Hangzhou 310009, China; ^e^Research Center for Life Science and Human Health, Binjiang Institute of Zhejiang University, Hangzhou 310053, China; ^f^Department of Surgical Oncology, Sir Run Run Shaw Hospital, Zhejiang University School of Medicine, Hangzhou 310016, China; ^g^Department of Respiratory Medicine, Sir Run Run Shaw Hospital, Zhejiang University School of Medicine, Hangzhou 310016, China; ^h^Zhejiang Key Laboratory of Precision Diagnosis and Therapy for Major Gynecological Diseases, Department of Gynecologic Oncology, Women’s Hospital, Zhejiang University School of Medicine, Hangzhou 310006, China

**Keywords:** CRL3-KCTD10, USP18, SLC7A11, ferroptosis, protein stability

## Abstract

SLC7A11 is a cystine transporter and ferroptosis inhibitor. How the stability of SLC7A11 is coordinately regulated in response to environmental cystine by which E3 ligase and deubiquitylase (DUB) remains elusive. Here, we report that neddylation inhibitor MLN4924 increases cystine uptake by causing SLC7A11 accumulation, via inactivating Cullin-RING ligase-3 (CRL-3). We identified KCTD10 as the substrate-recognizing subunit of CRL-3 for SLC7A11 ubiquitylation, and USP18 as SLC7A11 deubiquitylase. Upon cystine deprivation, the protein levels of KCTD10 or USP18 are decreased or increased, respectively, contributing to SLC7A11 accumulation. By destabilizing or stabilizing SLC7A11, KCTD10, or USP18 inversely regulates the cystine uptake and ferroptosis. Biologically, MLN4924 combination with SLC7A11 inhibitor Imidazole Ketone Erastin (IKE) enhanced suppression of tumor growth. In human breast tumor tissues, SLC7A11 levels were negatively or positively correlated with KCTD10 or USP18, respectively. Collectively, our study defines how SLC7A11 and ferroptosis is coordinately regulated by the CRL3^KCTD10^/E3-USP18/DUB axis, and provides a sound rationale of drug combination to enhance anticancer efficacy.

## Significance

SLC7A11 is a key determinant for cystine transport and ferroptosis, and is often dysregulated in many human diseases, including cancer. However, how the stability of SLC7A11 is regulated under physiological conditions, remains elusive. Here, we report that the SLC7A11 stability is regulated negatively by CRL3^KCTD10^ E3 ligase, and positively by USP18 deubiquitylase. The levels of KCTD10, SLC7A11, and USP18 are coordinately regulated by environment cystine. Through targeting SLC7A11, the KCTD10–USP18 axis regulates ferroptosis. Combination of inhibitors of Cullin-RING ligase-3 (CRL-3) and SLC7A11 enhances the antitumor efficacy. Our study reveals a regulatory mechanism of the SLC7A11 stability through the CRL3^KCTD10^/E3-USP18/DUB axis upon cystine starvation and provides a sound rationale of drug combination to enhance anticancer efficacy.

Cell death is essential for normal development and homeostasis maintenance, which is often dysregulated in a plethora of human diseases, including cancer (1, 2). A hallmark of cancer cells is their ability to evade cell death, which contributes to tumor development and therapeutic resistance (3). Among many types of cell death defined, ferroptosis, driven by iron-dependent lipid peroxidation, is morphologically, genetically, and biochemically unique (4, 5) with considerable attention, as its induction selectively kills certain types of cancer cells, offering the potential to overcome the resistance of cancer therapies (6).

Cystine, an oxidative cysteine dimer, was transported into cells via system X_c_^−^, a heterodimeric plasma membrane cystine/glutamate antiporter consisting of the transporter subunit solute carrier family 7 member 11 (SLC7A11) and the transmembrane regulatory subunit SLC3A2 (7). Once imported into cells, cystine is reduced to cysteine, which acts as the rate-limiting substrate for the biosynthesis of antioxidant glutathione (GSH), thus contributing to cellular redox homeostasis (8). GSH is subsequently utilized by glutathione peroxidase 4 (GPX4) to reduce lipid hydroperoxides to lipid alcohols, thus preventing lipid peroxidation in the cellular membranes, and eventually blocking ferroptosis (9). Given the cyst(e)ine/GSH/GPX4 axis is the mainstay defense system against ferroptosis (6), system X_c_^−^, especially SLC7A11, is regarded as a cardinal regulator of ferroptotic cell death (4). Although SLC7A11 plays an important role in cystine transport, and acts as a crucial determinant of ferroptosis, how SLC7A11 stability is coordinately regulated by which pairs of E3-deubiquitylase, and under what physiological and pathological conditions, is currently unknown.

Neddylation, an ubiquitylation-like posttranslational modification, is a biochemical reaction to attach NEDD8 onto the lysine residue of a substrate, which is catalyzed by NEDD8-activating enzyme (NAE/E1), NEDD8-conjugating enzyme (E2), and NEDD8 ligase (E3) (10). Cullin family proteins, the scaffold component of Cullin-RING ligase (CRL), are the best-characterized physiological substrates of neddylation (10). A typical CRL modular complex is composed of a scaffold cullin, an adaptor protein, a substrate-recognizing receptor, and a RING protein (10). Cullin neddylation activates CRLs, the largest family of E3 ubiquitin ligases that control many biological processes through ubiquitylation and degradation of many key signaling proteins (10).

Neddylation, which is overactivated in cancer and predictive of a poor clinical outcome (11), has been validated as a promising anticancer target (10). MLN4924, also known as pevonedistat, is the first-in-class small molecule inhibitor of NAE (12), currently in several phases I-III clinical trials for anticancer therapy as a single agent or in combination with chemo-drugs (10). Neddylation was also found to regulate various metabolic processes, including adipogenesis, lipid droplet formation, mitochondrial function, and cellular redox status (10, 13). Recently, we found that neddylation inactivation remarkably alters global metabolic profiling via inhibiting mitochondrial function and promoting glycolysis (14), and also induces glutamine uptake and metabolism by targeting CRL3^SPOP^ E3 ligase (15). Given that cystine metabolism via X_c_^−^ system orchestrates glutamine metabolism and sustains tumor cell growth (16, 17), it is likely that an undefined neddylation-CRL(s) system controls the levels and function of X_c_^−^ system to regulate ferroptosis.

Here, we reported that MLN4924 promotes cystine uptake by inactivating CRL3 to cause accumulation of SLC7A11. Mechanistically, CRL3^KCTD10^ is an E3 ligase, whereas USP18 is a DUB for SLC7A11, and the levels of the KCTD10–USP18 axis are coordinately regulated in response to environmental cystine for coordinated control of the SLC7A11 stability. Biologically, KCTD10 and USP18 confer breast cancer cells’ sensitivity or resistance to ferroptosis, respectively, via targeting SLC7A11. In breast cancer tissues with reduced cystine abundance, a negative correlation between KCTD10 and SLC7A11 and a positive correlation between USP18 and SLC7A11 were observed, respectively. Therapeutically, the combination of MLN4924 with SLC7A11 inhibitor Erastin or its derivative IKE markedly enhanced the killing of breast cancer cells both in vitro and in vivo. Collectively, we defined how the CRL3^KCTD10^/E3-USP18/DUB axis regulates ferroptosis by modulating SLC7A11 stability with translational implication by targeting both neddylation and SLC7A11 simultaneously for enhanced anticancer efficacy.

## Results

### Inactivation of CRL3 E3 Increases Cystine Uptake via SLC7A11 Accumulation.

**Fig. 1.**
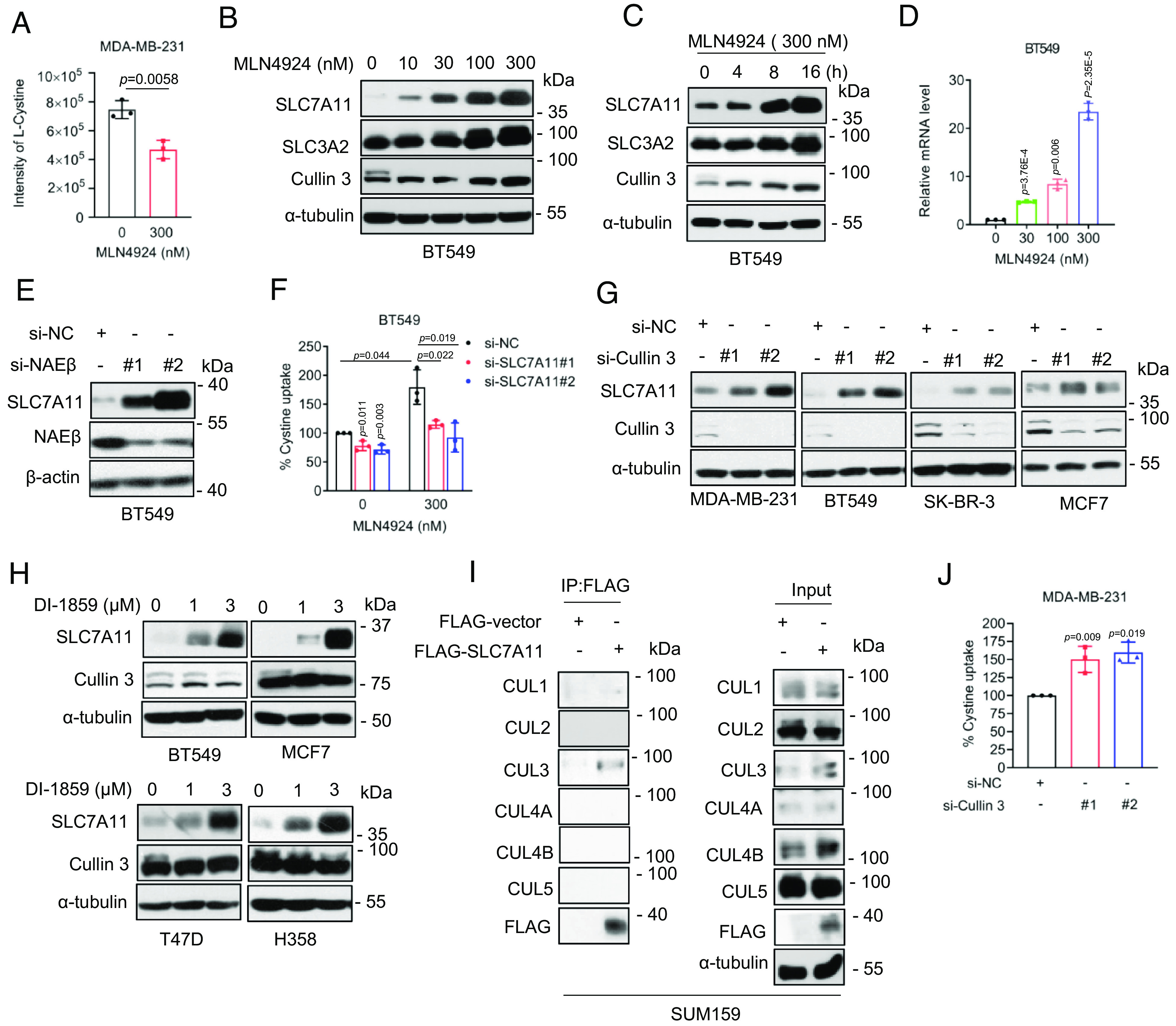
MLN4924 promotes cystine uptake through SLC7A11. (*A*) MDA-MB-231 cells were treated without or with 300 nM MLN4924 for 24 h and subjected to measurement of cystine levels in culture supernatant (mean ± SD, n = 3). (*B* and *C*) BT549 cells were treated with indicated concentrations of MLN4924 for 24 h (*B*) or indicated time points (*C*), analyzed by immunoblotting. (*D*) BT549 cells were treated with MLN4924 for 24 h, followed by qRT-PCR analysis (mean ± SD, n = 3). (*E*) BT549 cells were transfected with siRNAs (si-NC or si-NAEβs) for 48 h, followed by immunoblotting. (*F*) BT549 cells were transfected with indicated siRNAs (si-NC or si-SLC7A11s) for 24 h, then treated with 300 nM MLN4934, followed by cystine uptake detection after 24 h (mean ± SD, n = 3). (*G*) Indicated cancer cell lines were transfected with siRNAs (si-NC or si-Cullin 3 s) for 48 h, followed by immunoblotting. (*H*) Indicated breast cancer cell lines were treated with two concentrations of DI-1859 for 24 h, followed by immunoblotting. (*I*) SUM159 cells were transfected with a plasmid encoding FLAG-SLC7A11, followed by IP-IB analysis of the indicated proteins. (*J*) MDA-MB-231 cells were transfected with siRNAs (si-NC or si-Cullin 3 s) for 48 h, followed by cystine uptake detection (mean ± SD, n = 3).

We have recently shown that MLN4924 markedly changed global metabolisms, particularly glutamine metabolism, and significantly regulated the survival of breast cancer cells (14, 15). Given that environmental cystine and the X_c_^−^ system are actively involved in glutaminolysis (16, 17), a hallmark of cancer metabolism (18), we hypothesized that neddylation may regulate the glutamine metabolism through modulating the X_c_^−^ system. We first found that MLN4924 treatment lowered the levels of cystine in the culture supernatant (Fig. 1*A*), suggesting an activation of X_c_^−^ system for increased cystine uptake. We then found that after MLN4924 treatment, SLC7A11 levels were remarkably increased in time- and dose-dependent manners in multiple lines of cancer cells, while the SLC3A2 levels were increased moderately in a cell line–dependent manner (Fig. 1 *B* and *C* and *SI Appendix*, Fig. S1 *A*–*C*). We further used TAS4464, another selective inhibitor of NAE (19), and obtained similar results (*SI Appendix*, Fig. S1*D*). Moreover, MLN4924 significantly increased SLC7A11 mRNA levels (Fig. 1*D* and *SI Appendix*, Fig. S1*E*). Given both neddylation inhibitors robustly increased SLC7A11 levels, we focused on SLC7A11 for the rest of study.

We next knocked down NAEβ, the catalytic subunit of NAE to which MLN4924 inhibits, and found that the SLC7A11 levels were increased (Fig. 1*E* and *SI Appendix*, Fig. S1*F*), suggesting that SLC7A11 accumulation upon MLN4924 or TAS4464 treatment is an on-target effect. A rescuing experiment showed that SLC7A11 knockdown (*SI Appendix*, Fig. S1*G*) reduced MLN4924-induced cystine uptake (Fig. 1*F* and *SI Appendix*, Fig. S1*H*). Thus, enhanced cystine uptake by neddylation inactivation is likely via inducing SLC7A11 accumulation.

Remarkable accumulation of SLC7A11 by neddylation inhibitors strongly suggests that SLC7A11 is a substrate of CRLs, but which one? We, therefore, knocked down all five cullins (Cul-1, -2, -3, -4A, -4B, -5) individually, and found that only Cul-3 knockdown caused SLC7A11 accumulation in multiple breast cancer cell lines (Fig. 1*G* and *SI Appendix*, Fig. S1*I*). Consistently, treatment of breast and lung cancer cells with a specific Cul-3 inhibitor (DI-1859) (20) also caused SLC7A11 accumulation in a dose-dependent manner (Fig. 1*H*). The immunoprecipitation (IP) pull-down assay showed that SLC7A11 bound only to cullin-3 (Fig. 1*I* and *SI Appendix*, Fig. S1*J*). Finally, Cul-3 knockdown increased cystine uptake (Fig. 1*J* and *SI Appendix*, Fig. S1*K*). Thus, SLC7A11 appears to be a new substrate of CRL3 ligase, whose accumulation, upon CRL3 inactivation, is responsible for increased cystine uptake.

### KCTD10 Binds to and Negatively Regulates SLC7A11 Levels.

**Fig. 2.**
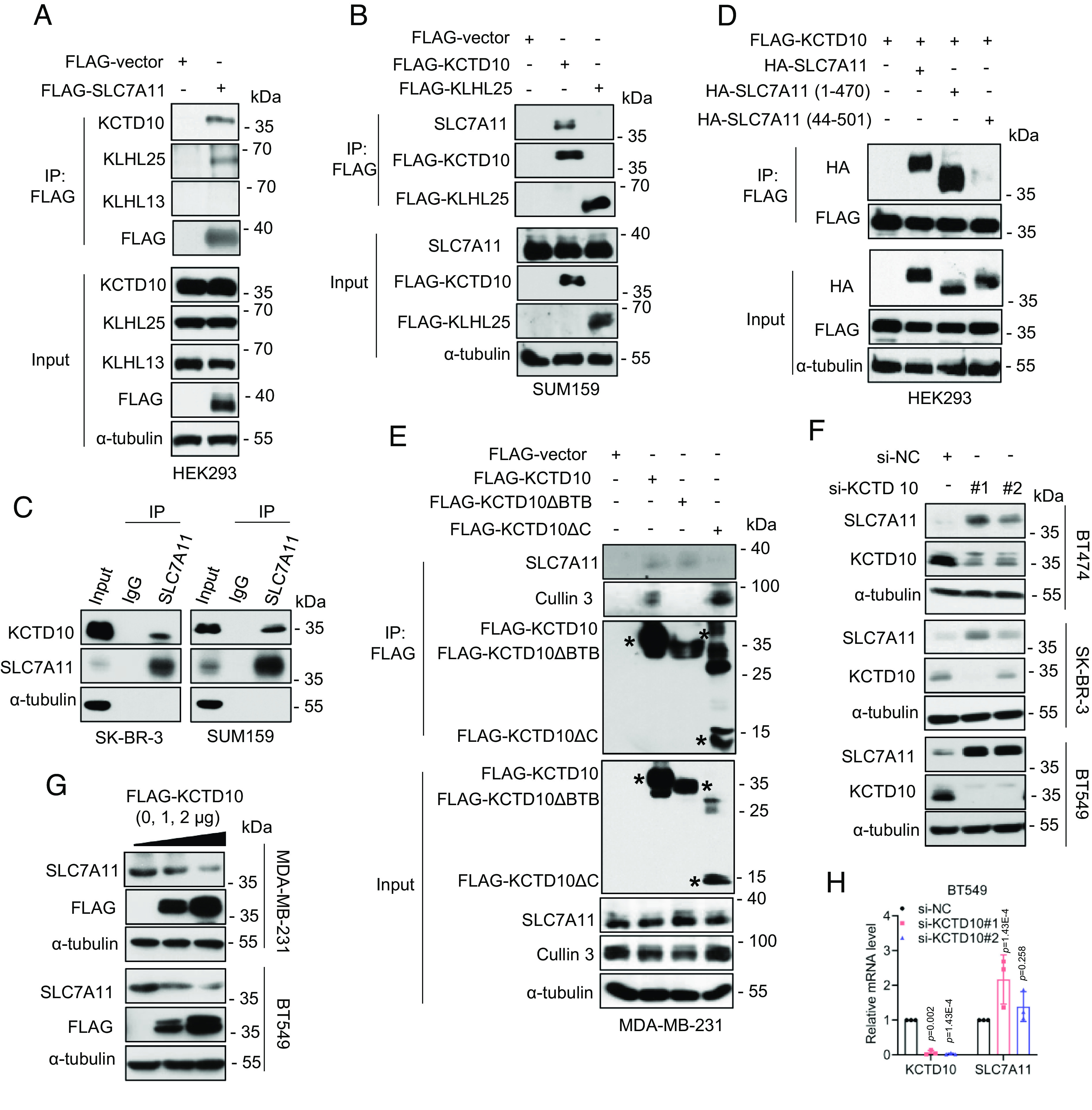
KCTD10 interacts with SLC7A11 and negatively regulates SLC7A11 levels. (*A*) HEK293 cells were transfected with a plasmid encoding FLAG-SLC7A11, followed by IP-IB analysis of the indicated proteins. (*B*) SUM159 cells were transfected with plasmids encoding FLAG-KCTD10 or FLAG-KLHL25, respectively, followed by IP-IB analysis of the indicated proteins. (*C*) IP-IB analysis of the indicated proteins under physiological condition. (*D*) HEK293 cells were transfected with indicated plasmids, followed by IP-IB analysis of the indicated proteins. (*E*) MDA-MB-231 cells were transfected with indicated plasmids, followed by IP-IB analysis of the indicated proteins. The asterisks indicate the corresponding proteins expressed. (*F*) Breast cancer cells were transfected with siRNAs (si-NC or si-KCTD10s) 48 h, followed by immunoblotting. (*G*) MDA-MB-231 and BT549 cells were transfected with increasing amounts of plasmids encoding KCTD10 for 48 h, followed by immunoblotting. (*H*) BT549 cells were transfected with siRNAs (si-NC or si-KCTD10s) for 48 h, followed by qRT-PCR analysis (mean ± SD, n = 3).

CRL3 ligase recognizes the downstream substrates through its substrate-receptor subunit (21). To identify the receptor subunit that is responsible for SLC7A11 binding, the siRNA-based knockdown approach was used. Given SLC7A11 is a potential oncoprotein, we selected 13 receptor proteins with known putative tumor suppressor function, and silenced them individually by a pool of three independent siRNAs. Knockdown of KLHL13, KLHL25, and KCTD10 increased the levels of SLC7A11 (*SI Appendix*, Fig. S2*A*). The IP-based pull-down assay showed that ectopically expressed SLC7A11 bound to endogenous KLHL25 and KCTD10, but not KLHL13 (Fig. 2*A*). In a reciprocal IP assay, ectopically expressed KCTD10, but not KLHL25, bound to endogenous SLC7A11 (Fig. 2*B*). Importantly, endogenous SLC7A11 bound to endogenous KCTD10 under physiological condition (Fig. 2*C*).

We next defined the SLC7A11-binding domain on KCTD10 by generating two SLC7A11 truncation mutants, MT-1: SLC7A11-1~470 and MT-2: SLC7A11-44~501 (*SI Appendix*, Fig. S2*B*) (22). A pull-down assay showed that wild-type SLC7A11 and MT-1, but not MT-2 bound to KCTD10 (Fig. 2*D*), indicating that the first 43 amino acids of SLC7A11 mediate KCTD10 binding. We then defined the KCTD10 binding domain on SLC7A11 by constructing two mutants: a BTB delete mutant (KCTD10ΔBTB) which fails to binds to Cul-3 and a C-terminal delete mutant (KCTD10ΔC), which fails to bind to substrate (23, 24) (*SI Appendix*, Fig. S2*B*). The IP-based pull-down assays showed that wildtype KCTD10 pulled down both endogenous SLC7A11 and Cul-3. KCTD10ΔBTB mutant pulled down endogenous SLC7A11, but not Cul-3, whereas the KCTD10ΔC mutant pulled endogenous Cul-3, but not SLC7A11 (Fig. 2*E*), indicating the C-terminal domain of KCTD10 is responsible for SLC7A11 binding. Collectively, the KCTD10–SLC7A11 binding is mediated by the N-terminal domain of SLC7A11 and the C-terminal domain of KCTD10 (*SI Appendix*, Fig. S2*B*). Although SLC7A11 is a membrane protein, its N-terminal domain is inserted into cytoplasm (25). The immunofluorescent staining showed that KCTD10 has both cytoplasmic and membrane localizations, and some of KCTD10 do colocalize with SLC7A11 (*SI Appendix*, Fig. S2*C*). Finally, we measured whether KCTD10 manipulation would actually change the levels of SLC7A11 after binding. Indeed, in both breast and colon cancer cells, KCTD10 knockdown caused the accumulation of endogenous SLC7A11 (Fig. 2*F* and *SI Appendix*, Fig. S2*D*), whereas KCTD10 overexpression reduced the levels of endogenous SLC7A11 in a dose-dependent manner (Fig. 2*G* and *SI Appendix*, Fig. S2*E*). Furthermore, KCTD10 knockdown had a minor, if any, effect on the SLC7A11 mRNA (Fig. 2*H* and *SI Appendix*, Fig. S2*F*). Collectively, CRL3^KCTD10^ ligase appears to interact with and negatively regulates SLC7A11 at the posttranslational levels.

### CRL3^KCTD10^ Is an E3 that Promotes SLC7A11 Ubiquitylation and Destabilization.

**Fig. 3.**
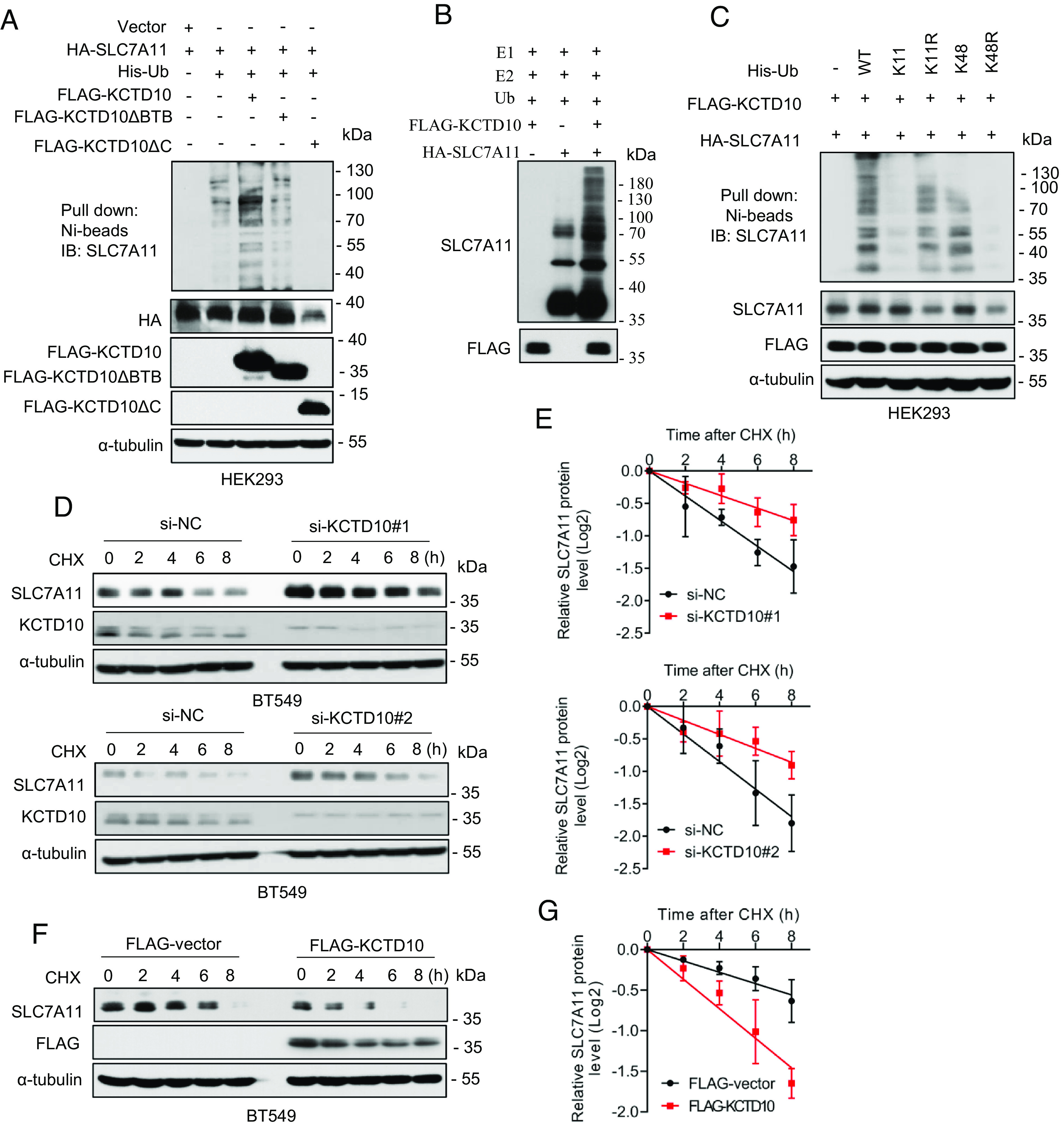
KCTD10 promotes SLC7A11 ubiquitylation and shortens SLC7A11 protein half-life. (*A*) HEK293 cells were transfected with indicated plasmids, followed by Ni-beads pulldown and immunoblotting for SLC7A11. (*B*) For in vitro ubiquitylation assay, purified HA-SLC7A11, as the substrate, was incubated with or without purified FLAG-KCTD10 in a reaction mixture containing E1 (UBE1), E2 (UbcH5a and Cdc34), ATP, and ubiquitin, followed by immunoblotting. (*C*) HEK293 cells were transfected with indicated plasmids, encoding wild type or mutant ubiquitins, followed by Ni-beads pulldown and immunoblotting for SLC7A11. (*D* and *E*) BT549 cells were transfected with siRNAs (si-NC or si-KCTD10s), then incubated with CHX for various time points before harvesting for immunoblotting (*D*), the band density of SLC7A11 was quantified and normalized to α-tubulin to draw a decay curve (mean ± SD, n = 3) (*E*). (*F* and *G*) BT549 cells were transfected with plasmid encoding FLAG-KCTD10, along with the vector control, then incubated with CHX for various time points before harvesting for immunoblotting (*F*), the band density of SLC7A11 was quantified and normalized to α-tubulin to draw a decay curve (mean ± SD, n = 3) (*G*).

We next used an in vivo ubiquitylation assay to determine whether CRL3^KCTD10^ ligase promoted SLC7A11 ubiquitylation. Indeed, KCTD10, but not KLHL25, promotes polyubiquitylation of exogenously expressed and endogenous SLC7A11 (*SI Appendix*, Fig. S3 *A* and *B*). Moreover, only wild-type KCTD10, but not its two mutants, promoted SLC7A11 polyubiquitylation (Fig. 3*A*). Consistently, in an in vitro ubiquitylation assay, SLC7A11 polyubiquitylation was clearly detected by CRL3^KCTD10^ E3 in the presence of purified ubiquitin, E1, and E2 (Fig. 3*B*). Finally, since the ubiquitin linkage via the K11 or K48 residue is doomed for substrate degradation, we used two sets of ubiquitin mutants with only K11 or K48 residue available or abrogated, and found that KCTD10-mediated SLC7A11 polyubiquitylation is via the K48 linkage (Fig. 3*C*).

Finally, we performed the protein half-life experiment and found that KCTD10 knockdown markedly extends, whereas KCTD10 overexpression shortens the protein half-life of endogenous SLC7A11 (Fig. 3 *D*–*G* and *SI Appendix*, Fig. S3 *C*–*F*). Collectively, KCTD10 destabilizes SLC7A11 by promoting its polyubiquitylation for subsequent proteasome degradation, leading to shortened protein half-life. Thus, SLC7A11 is a bona fide substrate of CRL3^KCTD10^ E3 ligase.

### USP18 Is a Deubiquitylase that Promotes SLC7A11 Deubiquitylation and Stabilization.

**Fig. 4.**
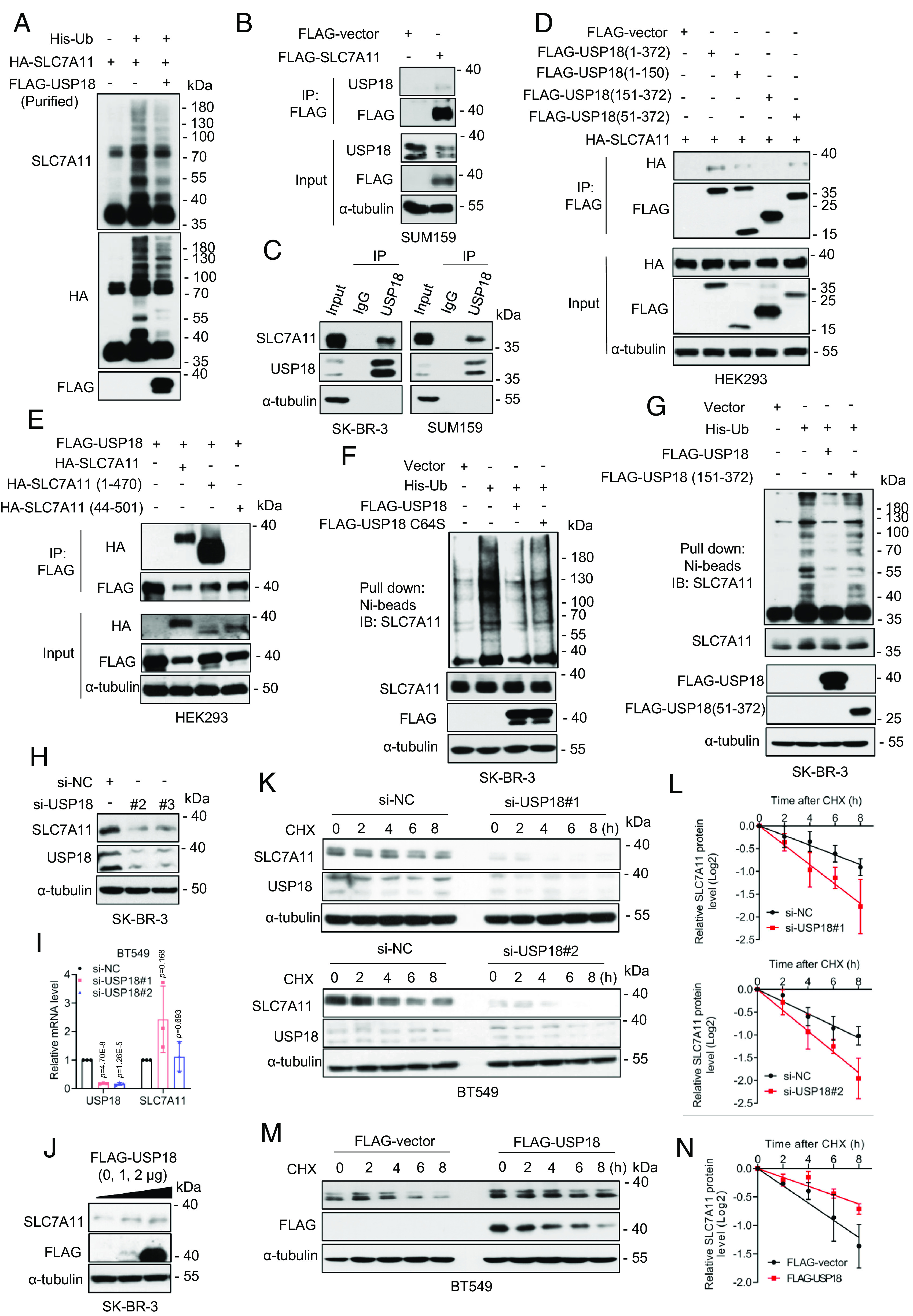
USP18 interacts with and deubiquitylates SLC7A11. (*A*) For in vitro deubiquitylation assay, HEK293T cells transfected with His-Ub and HA-SLC7A11. Ubiquitinated SLC7A11 was purified with IP using anti-HA Ab and was incubated without or with purified USP18, followed by immunoblotting. (*B*) SUM159 cells were transfected with a plasmid encoding FLAG-SLC7A11, followed by IP-IB analysis of the indicated proteins. (*C*) IP-IB analysis of the indicated proteins under physiological condition. (*D*) HEK293 cells were transfected with HA-SLC7A11 and indicated FLAG-tagged USP18 plasmids, followed by IP-IB analysis of the indicated proteins. (*E*) HEK293 cells were transfected with FLAG-USP18 and indicated HA-tagged SLC7A11 plasmids, followed by IP-IB analysis of the indicated proteins. (*F* and *G*) SK-BR-3 cells were transfected with indicated plasmids, followed by Ni-beads pulldown and immunoblotting for SLC7A11. (*H* and *I*) SK-BR-3 cells were transfected with siRNAs (si-NC or si-USP18s) for 48 h, followed by immunoblotting (*H*) and qRT-PCR analysis (mean ± SD, n = 3) (*I*). (*J*) SK-BR-3 cells were transfected with FLAG-USP18 plasmid, along with the vector control for 48 h, followed by immunoblotting. (*K* and *L*) BT549 cells were transfected with siRNAs (si-NC or si-USP18s), then incubated with CHX for various time points before harvesting for immunoblotting (*K*), the band density of SLC7A11 was quantified and normalized to α-tubulin to generate decay curve (mean ± SD, n = 3) (*L*). (*M* and *N*) BT549 cells were transfected with FLAG-USP18, along with the vector control, then incubated with CHX for various time points before harvesting for immunoblotting (*M*), the band density of SLC7A11 was quantified and normalized to α-tubulin to generate decay curve (mean ± SD, n = 3) (*N*).

The level of a given protein is dynamically regulated by an E3 ubiquitin ligase for destabilization, and a deubiquitylase for stabilization (26). To define exactly how the stability of SLC7A11 is regulated, we aimed to identify the DUBs responsible for SLC7A11 binding and stabilization. An IP-based pull-down assay after transient transfection of a battery of DUBs showed that USP2, USP3, USP8, USP18, and USP21 interacted with endogenous SLC7A11 (*SI Appendix*, Fig. S4*A*). We then determined which of these DUBs could remove the ubiquitin chain from SLC7A11, and found that USP18 is the only one that significantly reduced SLC7A11 polyubiquitylation (*SI Appendix*, Fig. S4*B*). Moreover, in an in vitro deubiquitylation assay, purified USP18 protein significantly removed polyubiquitylated chains of SLC7A11 (Fig. 4*A*). Thus, we focused on USP18 for further detailed characterization.

We first validated the interaction between SLC7A11 and USP18, and found that ectopically expressed SLC7A11 interacts with endogenous USP18 (Fig. 4*B*). Moreover, the interaction between SLC7A11 and USP18 at endogenous levels was also detected (Fig. 4*C*). We next made a series of UPS18 deletion mutants (27) to map the region that mediates SLC7A11 binding, and found that the internal domain of USP18 (AA 51-150) is required for its interaction with SLC7A11 (Fig. 4*D* and *SI Appendix*, Fig. S4*C*), whereas the N-terminal domain of SLC7A11 (AA 1-43) is responsible for its binding with USP18 (Fig. 4*E* and *SI Appendix*, Fig. S4*C*). The immunofluorescent staining showed that USP18 has both cytoplasmic and membrane localizations, and colocalization of USP18 with SLC7A11 was detectable (*SI Appendix*, Fig. S4*D*).

We next examined the DUB activity of USP18 on SLC7A11, and found that wild-type USP18, but not the enzyme-dead mutant USP18-C64S (27) nor USP18 (151-372) mutant with the SLC7A11 binding domain-deleted, markedly removed polyubiquitylated chains of SLC7A11 (Fig. 4 *F* and *G* and *SI Appendix*, Fig. S5 *A* and *B*).

Finally, we determined whether USP18 manipulations would affect SLC7A11 levels and its protein half-life. Indeed, USP18 knockdown significantly reduced SLC7A11 levels in multiple human cancer lines (Fig. 4*H* and *SI Appendix*, Fig. S5*C*), but had moderate effects on SLC7A11 mRNA levels (Fig. 4*I* and *SI Appendix*, Fig. S5*D*). Moreover, USP18 overexpression increased SLC7A11 levels in a dose-dependent manner (Fig. 4*J* and *SI Appendix*, Fig. S5*E*). Consistently, USP18 knockdown shortened, and USP18 overexpression extended the protein half-life of SLC7A11 (Fig. 4 *K*–*N* and *SI Appendix*, Fig. S5 *F*–*I*), respectively. To further confirm the role of USP18 in regulating SLC7A11 turnover, we generated USP18 knockout MDA-MB-231 cells through the CRISPR-Cas9 approach, and found that USP18 deletion decreased SLC7A11 levels (*SI Appendix*, Fig. S5*J*), shortened its protein half-life (*SI Appendix*, Fig. S5 *K* and *L*), and increased the polyubiquitylation of SLC7A11 (*SI Appendix*, Fig. S5*M*). Taken together, USP18 appears to be a bona fide DUB for SLC7A11 stabilization at posttranslational levels.

### Cystine Regulates SLC7A11 Levels and Cell Viability via the KCTD10–USP18 Axis.

We next determined under what physiological or stress conditions that SLC7A11, KCTD10, and USP18 are coordinately regulated. Given that the change of SLC7A11 level is associated with glutamine metabolism (16, 17), we first measured the levels of these three proteins under glutamine deprivation conditions, and found that glutamine deprivation caused a time-dependent increase in the levels of SLC7A11 and USP18 (in a cell line–dependent manner), but had no effect on KCTD10 (*SI Appendix*, Fig. S6 *A* and *B*, *Left*). SLC7A11 was reported to be subjected to glucose upregulation (28), we then measured possible glucose regulation of these three proteins. Glucose deprivation increased the levels of SLC7A11 and USP18, but had no effect on the levels of KCTD10 (*SI Appendix*, Fig. S6 *A* and *B*, *Right*). Thus, unlike KCTD10, SLC7A11 and USP18 (to a lesser extent) are subjected to regulation by stresses, such as deprivation of glutamine and glucose.

**Fig. 5.**
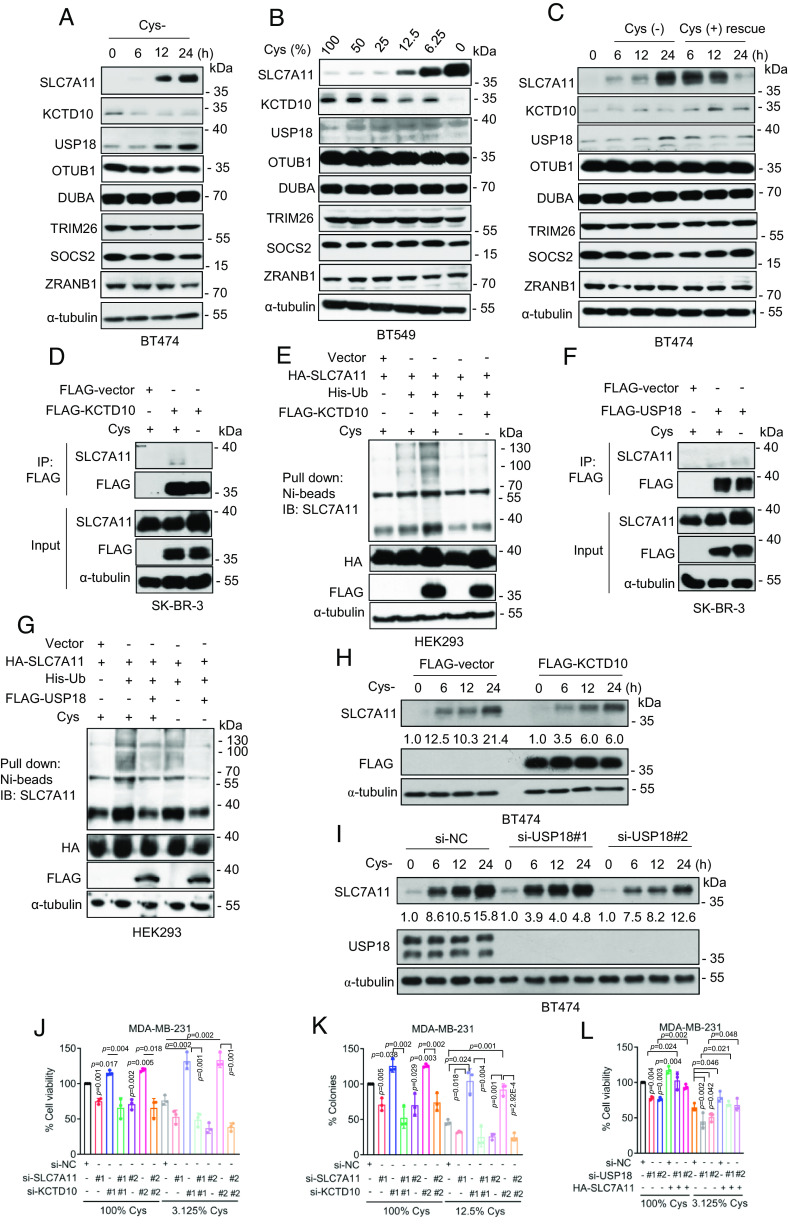
Cystine deprivation regulates KCTD10- and USP18-mediated ubiquitylation of SLC7A11. (*A*) BT474 cells were cultured with cystine-free medium for different time points, followed by immunoblotting. (*B*) BT549 cells were cultured with indicated percentage of cystine for 24 h, followed by immunoblotting. (*C*) BT474 cells were cultured with cystine-free medium for different time points, followed by cystine addition at 24 h time-point for different time points, followed by immunoblotting. (*D*) SK-BR-3 cells were transfected with FLAG-KCTD10 plasmid, along with the vector control, cultured in cystine-containing or cystine-free media for 24 h, followed by IP-IB analysis of the indicated proteins. (*E*) HEK293 cells were transfected with indicated plasmids, cultured in cystine-containing or cystine-free media for 24 h, followed by Ni-beads pulldown, immunoblotting for SLC7A11. (*F*) SK-BR-3 cells were transfected with FLAG-USP18 plasmid, along with the vector control, cultured in cystine-containing or cystine-free media for 24 h, followed by IP-IB analysis of the indicated proteins. (*G*) HEK293 cells were transfected with indicated plasmids, cultured in cystine-containing or cystine-free media for 24 h, followed by Ni-beads pulldown, immunoblotting for SLC7A11. (*H*) BT474 cells were transfected with FLAG-KCTD10 plasmid, along with the vector control for 48 h, then cultured with cystine-free medium for different time points, followed by immunoblotting. (*I*) BT474 cells were transfected with siRNAs (si-NC or si-USP18s) for 48 h, then cultured with cystine-free medium for different time points, followed by immunoblotting. (*J* and *K*) MDA-MB-231 cells were transfected with indicated siRNAs and cultured with medium containing 100% and 3.125% cystine, respectively, then cell viability (*J*) and clonogenic survival (*K*) were measured (mean ± SD, n = 3). (*L*) MDA-MB-231 cells were transfected with indicated siRNAs and plasmids, cultured with medium containing 100% and 3.125% cystine, respectively, for 48 h, then cell viability was measured (mean ± SD, n = 3).

Given SLC7A11 plays a major role of cystine transportation and was subjected to cystine regulation (29), we next focused mainly on cystine regulation on these three proteins. Strikingly, complete cystine deprivation increased the levels of SLC7A11 and USP18, but decreased the levels of KCTD10 in a time-dependent manner (Fig. 5*A*). Moreover, the levels of SLC7A11 and USP18 were negatively correlated, whereas the levels of KCTD10 were positively correlated with the levels of cystine in the culture media, respectively (Fig. 5*B* and *SI Appendix*, Fig. S6 *C* and *D*). Furthermore, the increase of SLC7A11 and USP18 and decrease of KTD10 proteins induced by cystine deprivation could be largely rescued by cystine resupply in a time-dependent manner (Fig. 5*C* and *SI Appendix*, Fig. S6*E*). Interestingly, we found that TRIM26, SOCS2, and ZRANB1, three E3s reported to regulate SLC7A11 in liver cancer cell lines (30), mouse liver (31), or kidney renal clear cell carcinoma (32), and OTUB1 and DUBA, two DUBs, reported to regulate SLC7A11 in few types of human cancer cell lines (22) or differentiated cancer stem cells (33), respectively, were not subjected to cystine regulation in breast cancer cells (Fig. 5 *A*–*C* and *SI Appendix*, Fig. S6 *C*–*E*). Thus, increased USP18 protein levels coupled with decreased KCTD10 protein levels in response to environmental cystine starvation ensure the high levels of SLC7A11 to enhance cellular uptake of cystine.

We further investigated whether cystine deprivation affects the interaction of SLC7A11 with KCTD10 or with USP18. Significantly, cystine deprivation caused dissociation of SLC7A11 with KCTD10 (Fig. 5*D* and *SI Appendix*, Fig. S6*F*), and inhibited SLC7A11 polyubiquitylation induced by KCTD10 (Fig. 5*E*). On the other hand, cystine deprivation enhanced the SLC7A11–USP18 interaction (Fig. 5*F* and *SI Appendix*, Fig. S6*G*), and promoted SLC7A11 deubiquitylation induced by USP18 (Fig. 5*G*). Furthermore, under the cystine-deprived condition, either KCTD10 overexpression or USP18 knockdown delayed the process of SLC7A11 accumulation (Fig. 5 *H* and *I* and *SI Appendix*, Fig. S6 *H* and *I*), further suggesting that SLC7A11 levels induced upon cystine deprivation are subjected to regulation by KCTD10 and USP18. Taken together, the levels and interaction among SLC7A11, KCTD10, and USP18 are coordinately regulated in response to cystine starvation to ensure the highest level of SLC7A11 for effective cystine uptake.

Finally, we performed biological assays to determine whether KCTD10 or USP18 coordinates with SLC7A11 to regulate the cell viability and clonogenic survival of breast cancer cells. Under either cystine-enriched or cystine-starved condition, SLC7A11 knockdown inhibited, whereas KCTD10 knockdown promoted cell viability and clonogenic survival (Fig. 5 *J* and *K* and *SI Appendix*, Fig. S6 *J* and *K*), suggesting that SLC7A11 promotes cell viability and survival, whereas KCTD10 inhibits them in breast cancer cells. Interestingly, coknockdown of both SLC7A11 and KCTD10 also suppressed cell viability and clonogenic survival (Fig. 5 *J* and *K* and *SI Appendix*, Fig. S6 *J* and *K*), suggesting SLC7A11 plays a major role. Likewise, USP18 knockdown inhibited cell viability, which was rescued by ectopic SLC7A11 expression (Fig. 5*L* and *SI Appendix*, Fig. S6*L*), suggesting the survival-promoting role of USP18 is mediated via up-regulating SLC7A11. Collectively, regulation of cell viability and survival by KCTD10 and USP18 is centered in targeting SLC7A11.

### The KCTD10–USP18 Axis Regulates Cell Viability via Ferroptosis by Targeting SLC7A11.

**Fig. 6.**
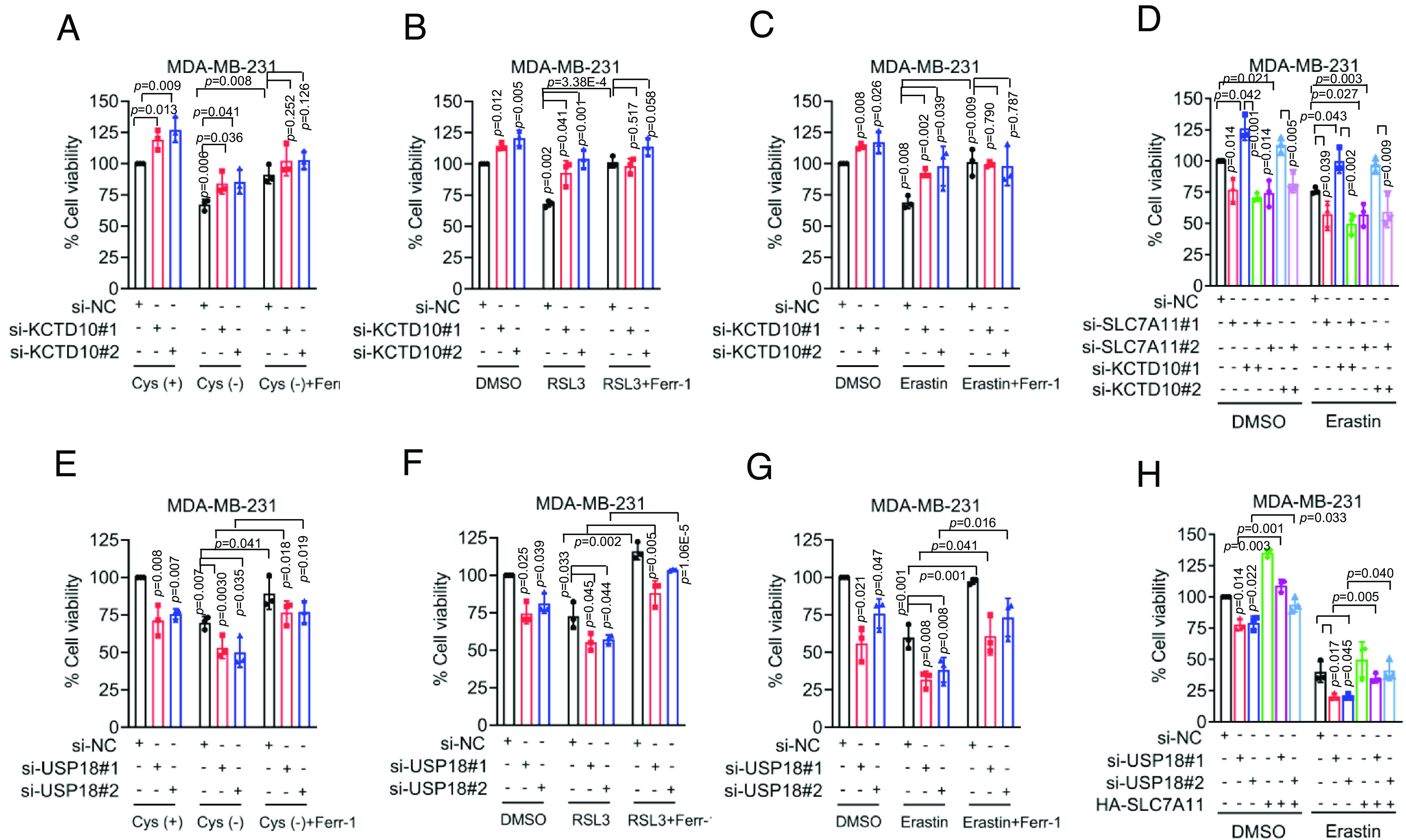
KCTD10 and USP18 regulate ferroptosis through SLC7A11. (*A*–*C*) MDA-MB-231 cells were transfected with indicated siRNAs (si-NC or si-KCTD10s), followed by cystine deprivation (*A*), treatment with RSL3 (*B*), and Erastin (*C*) alone or in combination with Ferr-1 for 24 h as indicated, then cell viability was measured (mean ± SD, n = 3). (*D*) MDA-MB-231 cells transfected with indicated siRNAs (si-NC or si-KCTD10s) for 24 h, then treated without and with Erastin for 48 h, followed by cell viability measurement (mean ± SD, n = 3). (*E*–*G*) MDA-MB-231 cells were transfected with indicated siRNAs (si-NC or si-USP18s), followed by cystine deprivation (*E*), treatment with RSL3 (*F*) and Erastin (*G*) alone or together with Ferr-1 for 24 h, as indicated, followed by cell viability measurement (mean ± SD, n = 3). (*H*) MDA-MB-231 cells transfected with indicated siRNAs (si-NC or si-USP18s) and plasmid (HA-SLC7A11) for 24 h, then treated without and with Erastin for 48 h, followed by cell viability measurement (mean ± SD, n = 3).

To pursue the nature of cell viability regulation, we focused on ferroptosis, which is regulated by SLC7A11 (4, 34). We first confirmed that KCTD10 knockdown significantly promoted cystine uptake (*SI Appendix*, Fig. S7 *A* and *B*), indicating that KCTD10 inhibits cystine transport via targeting SLC7A11 for degradation. We then determined whether ferroptosis induced by cystine deprivation (34, 35) is regulated by KCTD10. Indeed, cystine deprivation significantly suppressed cell viability via ferroptosis, which was reversed by ferroptosis inhibitor Ferrostatin-1 (Ferr-1) (36) (Fig. 6*A* and *SI Appendix*, Fig. S7*C*), as well as by KCTD10 knockdown (Fig. 6*A* and *SI Appendix*, Fig. S7*C*). KCTD10 knockdown also rescued viability inhibition induced by RSL3, a small molecule that induces ferroptosis through binding to and inactivating GPX4 (9), and this effect was blocked by Ferr-1 (Fig. 6*B* and *SI Appendix*, Fig. S7*D*). Similar rescue effect of KCTD10 knockdown were also observed when cells were treated with Erastin, a small molecule that induces ferroptosis by inhibiting cystine/glutamate transporter SLC7A11 (4), which was also blocked by Ferr-1 (Fig. 6*C* and *SI Appendix*, Fig. S7*E*). Finally, we validated that KCTD10 suppression of ferroptosis was through targeting SLC7A11 by knocking down KCTD10 or SLC7A11, individually, or in combination. Indeed, ferroptotic inhibition by KCTD10 knockdown was largely rescued by simultaneous SLC7A11 knockdown, and this effect was further enhanced by Erastin treatment (Fig. 6*D* and *SI Appendix*, Fig. S7 *F* and *G*). Collectively, these results demonstrated that the KCTD10 regulation of cell viability is via modulating ferroptosis by targeting SLC7A11.

We next investigated whether USP18 also affected the cell viability of breast cancer cells via modulating ferroptosis. We first confirmed that USP18 knockdown indeed reduced cystine uptake (*SI Appendix*, Fig. S7 *H* and *I*). Significantly, USP18 knockdown sensitized breast cancer cells to ferroptosis, induced by cystine deprivation, treatment with RSL3, or Erastin, which was all rescued by Ferr-1 (Fig. 6 *E*–*G* and *SI Appendix*, Fig. S7 *J*–*L*). We further confirmed that USP18 modulation of ferroptosis was through SLC7A11, since ectopic SLC7A11 expression largely rescued ferroptotic cell death induced by Erastin and enhanced by USP18 knockdown (Fig. 6*H* and *SI Appendix*, Fig. S7*M*).

### The Correlation between SLC7A11 and the KCTD10–USP18 Axis in Breast Cancer Tissues.

**Fig. 7.**
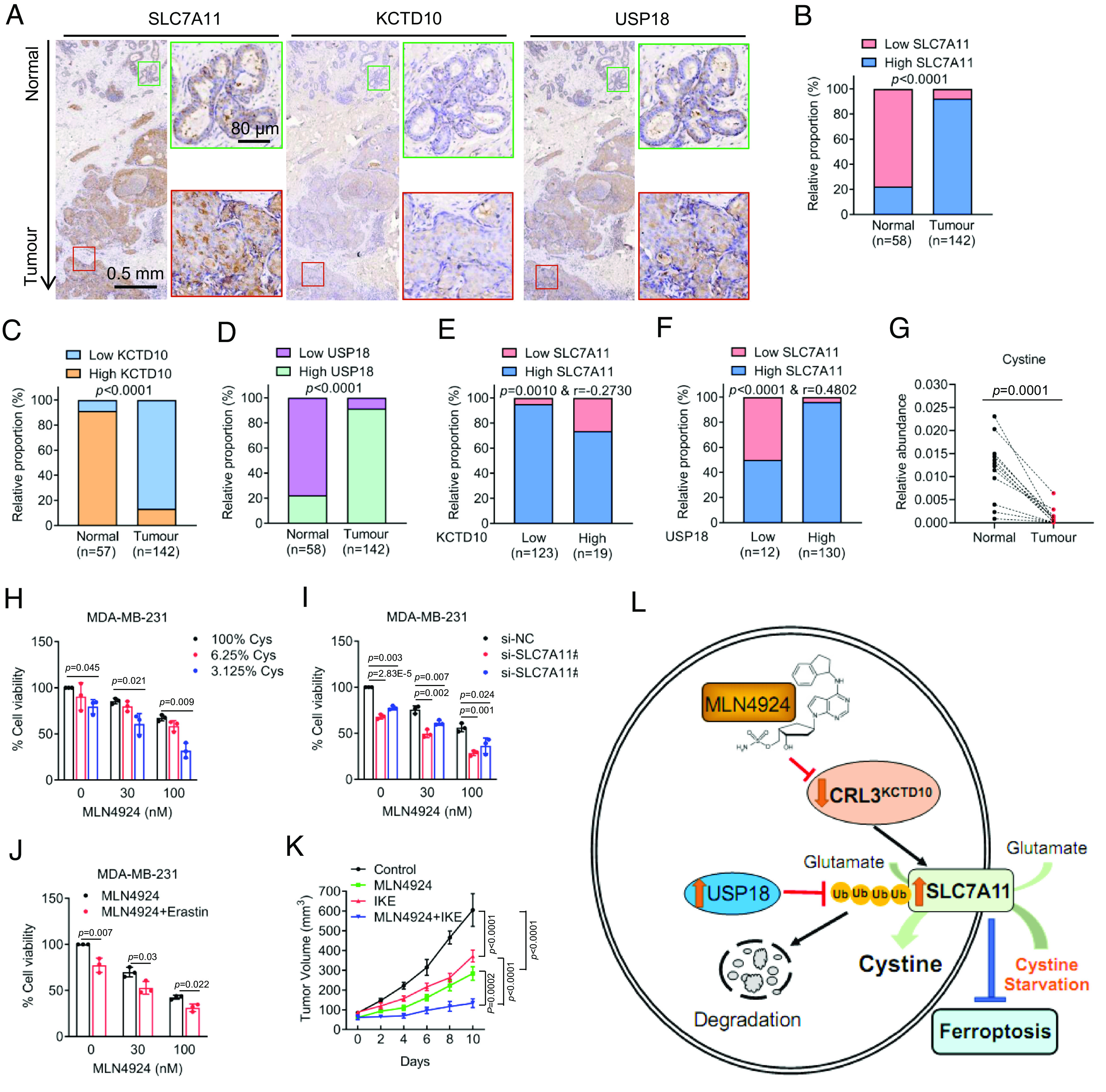
Correlation between SLC7A11 and the KCTD10–USP18 axis, and cotargeting neddylation and SLC7A11 enhances cancer killing. (*A*) Representative staining images of SLC7A11, KCTD10, and USP18 in consecutive breast tissues (normals vs. tumors). The green and red boxes indicate normal and tumor regions, respectively. (*B*–*D*) The relative proportion of the levels of SLC7A11 (*B*), KCTD10 (*C*), and USP18 (*D*) by IHC staining in adjacent nontumor and tumor tissues of the breast. (*E* and *F*) The correlation analysis of the staining levels between KCTD10 and SLC7A11 (*E*) as well as USP18 and SLC7A11 (*F*) in breast tumor sections. (*G*) Paired human breast normal and tumor tissues were analyzed by targeted metabolomics, and relative abundance of cystine was compared between each individual pair of normal vs. tumor tissues (n = 12). (*H*) MDA-MB-231 cells were treated with MLN4924 under various percentage of cystine in culture media for 48 h, followed by cell viability measurement (mean ± SD, n = 3). (*I*) MDA-MB-231 cells were transfected with siRNAs (si-NC or si-SLC7A11s) and then treated with DMSO control or MLN4924 for 48 h, followed by cell viability measurement (mean ± SD, n = 3). (*J*) MDA-MB-231 cells were treated with different concentrations of MLN4924 without or with Erastin for 48 h, followed by cell viability measurement (mean ± SD, n = 3). (*K*) The in vivo growth of MDA-MB-231 xenograft tumors after treatment of MLN4924, or IKE, alone or in combination, along with vehicle control for 14 d. Tumor growth was monitored (mean ± SEM, n = 6). (*L*) A working model: See text for the detail.

We next searched the Cancer Genome Atlas (TCGA) database for altered expression of SLC7A11, KCTD10, and USP18 in human cancer tissues. Compared to normal tissues, expression of SLC7A11 and USP18 was increased, whereas expression of KCTD10 was decreased in multiple types of human cancers, including breast cancer (*SI Appendix*, Fig. S8 *A*–*G*). The IHC staining of human breast cancer tissues showed that in general, the SLC7A11 levels were positively correlated with USP18 levels, but negatively correlated with the KCTD10 levels in both nontumor and tumor tissues (Fig. 7*A*). Consistently, the IHC-staining of a breast tumor tissue microarray, containing large number of tumor tissues (n = 142) and the adjacent normal tissues (n = 58) showed that compared to adjacent normal tissues, tumor tissues had significantly higher levels of SLC7A11 and USP18, but low levels of KCTD10 (Fig. 7 *B*–*D* and *SI Appendix*, Fig. S8*H*). Furthermore, in 123 cases with low-level of KCTD10, greater than 95% of cases had high SLC7A11 (Fig. 7*E*), whereas in 130 cases with high-level of USP18, more than 96% of cases had high SLC7A11 (Fig. 7*F*). Thus, high levels of E3 ligase (KCTD10) are correlated with low levels of its substrate (SLC7A11), whereas it is just opposite in case of DUB (USP18) with its substrate.

Finally, we analyzed our recent results of LC-MS/MS-based targeted metabolomics between breast normal and tumor tissues (15). In paired samples from the same patient, the levels of cystine were significantly lower in cancer tissues than in normal tissues (Fig. 7*G* and *SI Appendix*, Fig. S8*I*). Increased SLC7A11 levels (increased cysteine transport), coupled with reduced cystine levels in tumor tissues highly suggested an enhanced cystine metabolism. Collectively, this correlation study suggested that high levels of SLC7A11, likely as the consequence of low levels of KCTD10 and high levels of USP18 in cancer tissues, would trigger enhanced cystine transport to facilitate rapid cystine metabolism, eventually leading to cystine addiction in breast cancer cells.

### Enhanced Therapeutic Efficacy in Combination of MLN4924 and SLC7A11 Inhibitors.

As SLC7A11 accumulation by MLN4924 increased cystine uptake (Fig. 1), we then determined whether increased cystine uptake regulates cell survival. Indeed, under neddylation blocked condition, cystine starvation enhanced inhibition of cell viability dependent of the doses of both MLN4924 and cystine (Fig. 7*H* and *SI Appendix*, Fig. S8*J*). Moreover, SLC7A11 knockdown further enhanced inhibition of cell viability by MLN4924 (Fig. 7*I* and *SI Appendix*, Fig. S8*K*). Thus, it appears that SLC7A11 accumulation by MLN4924 promoted cystine uptake to support breast cancer survival, which would compromise the anticancer activity of MLN4924.

We next tested our working hypothesis that SLC7A11 inhibitor Erastin would sensitize breast cancer cells to MLN4924. Indeed, in the cell culture setting, Erastin increased inhibitory effects of MLN4924 (Fig. 7*J* and *SI Appendix*, Fig. S8*L*). In an in vivo xenograft model of MDA-MB-231 cells with MLN4924 alone or in combination with IKE, an Erastin derivative (37) at nontoxic doses (*SI Appendix*, Fig. S8*M*), we observed that compared to the vehicle control, tumor growth was significantly inhibited by MLN4924 or IKE alone, and with maximal inhibition by the combination, as evidenced by growth rate, and remarkable reduction in tumor size and weight (Fig. 7*K* and *SI Appendix*, Fig. S8 *N* and *O*). Thus, the combination of neddylation inhibitor MLN4924 and SLC7A11 inhibitor IKE would be a strategic choice for effective therapy against breast cancer.

Given breast tumor growth was significantly suppressed in an in vivo xenograft model following neddylation blockage and SLC7A11 inhibition, it promoted us to determine whether targeting KCTD10 or USP18 in combination with SLC7A11 inhibition could similarly affect tumor growth in this model. Given no KCTD10 activators or USP18 inhibitors were reported, the genetic approaches were used to knockdown/knockout KCTD10 or USP18, respectively. Stable cells were implanted into nude mice, followed by the treatment of SLC7A11 inhibitor IKE. Consistent with the in vitro cell culture model, tumors with KCTD10 knockdown (*SI Appendix*, Fig. S8*P*) are more resistance to IKE-induced growth inhibition (*SI Appendix*, Fig. S8*Q*), whereas tumors with USP18 knockout (*SI Appendix*, Fig. S5*J*) are more sensitive (*SI Appendix*, Fig. S8*R*). Collectively, KCTD10 has growth-suppressive, while USP18 has growth-promoting role in breast cancer cells. Combination of KCTD10 activation or USP18 inactivation with SLC7A11 inhibition appears to be an effective approach to inhibit tumor growth.

## Discussion

In this study, we showed that in addition to its role in regulation of mitochondrial energy reprograming (14) and glutamine metabolism (15), MLN4924 also altered cystine metabolism by increasing cystine uptake via inactivating CRL3 E3 ligase to cause accumulation of SLC7A11, a cystine transporter. We further characterized that SLC7A11 as a substrate of CRL3^KCTD10^ E3 ligase and USP18 deubiquitylase, and the SLC7A11 stability is precisely regulated by this E3-DUB pair in response to environmental cystine.

SLC7A11 is a key regulator of ferroptosis, which are associated with various pathological conditions (35). SLC7A11 level is subjected to regulation at the multiple layers. At the transcriptional levels, SLC7A11 is up-regulated by ATF4, NRF2, and TEAD-dependent YAP/TAZ but down-regulated by p53 (38–40). At the posttranscriptional levels, SLC7A11 is up-regulated by LncRNA HEPFAL and down-regulated by BAP1 and m^6^A reader YTHDC2 (41–43). At the posttranslational levels, SLC7A11 activity is negatively regulated by SLC7A11 phosphorylation at Ser26 residue, mediated by mTORC2 (44). At the posttranslational level, SLC7A11 levels are regulated by the ubiquitin–proteasome system (UPS). Specifically, SLC7A11 was reported to be a substrate of CRL5^SOCS2^ E3 ligase (30) or of TRIM26 E3 ligase (31). Interestingly, a recent study showed that ZRANB1, a deubiquitinase acts as an E3 ubiquitin ligase for SLC7A11 ubiquitylation (32). On the other hand, OTUB1 (22) and DUBA (33) were two deubiquitylases reported to stabilize SLC7A11. However, the characterizations of these SLC7A11 E3s and DUBs were mainly based upon overexpression approaches. It is unknown under what physiological or pathological conditions in which SLC7A11 is subjected to regulation by these E3s or DUBs.

Here, we reported that CRL3^KCTD10^ E3 ligase is responsible for SLC7A11 ubiquitylation in response to environmental cystine levels in breast cancer cells. Our conclusion is supported by the following lines of evidence: 1) pharmacological inhibition of neddylation or siRNA-based knockdown of Cul-3 causes SLC7A11 accumulation; 2) KCTD10 binds to SLC7A11 via its C-terminal region and SLC7A11 interacts with KCTD10 via its N-terminal domain; 3) KCTD10 overexpression or knockdown decreases or increases SLC7A11 levels, respectively; 4) KCTD10 overexpression or knockdown shortens or extends SLC7A11 protein half-life, respectively; 5) KCTD10 promotes SLC7A11 ubiquitylation for proteasome degradation. Thus, our study adds SLC7A11 to the repertoire of the growing list of KCTD10 substrates (23).

It was reported that KCTD10 interacts and promotes polyubiquitylation of its substrates via either K27 linkage for degradation of EIF3D and TRIF (45, 46), or the K63 linkage in RhoB for modulating its subcellular localization, activity, and lysosomal degradation (47). Our study has, however, revealed that KCTD10 utilizes the K48-linkage for SLC7A11 polyubiquitylation, a canonical linkage type for proteasome degradation (48). Furthermore, it is known that CRL3^Keap1^ and SCF^β-TrCP^ are two E3 ligases for targeted ubiquitylation and degradation, respectively, of NRF2 and ATF4 (49, 50), two transcription factors known to transactivate SLC7A11 mRNA expression (39, 40). Inactivation of CRL E3 ligase through neddylation blockage (by MLN4924) would, therefore, cause the accumulation of SLC7A11 at the transcriptional levels by the Keap1-NRF2-SLC7A11 and β-TrCP-ATF4-SLC7A11 axes through increased transcription, and at the posttranslational level by the KCTD10–SLC7A11 axis through reduced degradation.

In this study, we also characterized USP18 as a deubiquitylase that stabilizes SLC7A11 with the following lines of supporting evidence: 1) USP18 binds to SLC7A11 through its intermediate domain (AA 51-150) and SLC7A11 interacts with USP18 through its N-terminal domain; 2) USP18 overexpression or knockdown increases or decreases SLC7A11 levels, by extending or shortening the SLC7A11 protein half-life, respectively; 3) USP18 decreases the levels of SLC7A11 polyubiquitylation.

Whether or under what physiological and pathological conditions that SLC7A11 is subjected to KCTD10 and USP18 regulation? Here we showed a dynamic regulation of the SLC7A11 stability by KCTD10 and USP18 in response to cystine levels in the culture, as evidenced by 1) cystine deprivation decreases the levels of KCTD10, but increases the levels of USP18, which is reversed/rescued by cystine resupply, and 2) cystine deprivations disrupts the SLC7A11–KCTD10 interaction to reduce SLC7A11 ubiquitylation, whereas increases the SLC7A11–USP18 interaction to enhance SLC7A11 deubiquitylation. Importantly, none of previously reported SLC7A11 E3s (TRIM26, SOCS2, and ZRANB1) nor DUBs (OTUB1 and DUBA) are subjected to cystine regulation (Fig. 5).

What is the biological significance of coordinated regulation of the SLC7A11 stability by KCTD10 and USP18, particularly upon cystine starvation? We propose that during breast carcinogenesis, decreased KCTD10 and increased USP18 would cause SLC7A11 accumulation to increase cystine uptake and consumption for the maintenance of rapid metabolism. Consistently, the high levels of SLC7A11 and USP18, and low levels of KCTD10 are coordinately coupled with decreased cystine levels in breast cancer tissues (Fig. 7), indicating the pathological relevance. While SLC7A11 is up-regulated in diverse tumors which predicts adverse outcomes of patients (8), the underlying mechanisms by which cancer cells adapt to increased SLC7A11 levels remain largely unknown. Our study here elucidated, at least in part, the mechanism by which some types of cancer, including breast cancer, adjust elevated SLC7A11 levels to meet a high requirement of cystine consumption, and subsequent cysteine-depleted microenvironment to ensure their survival (51). Taken together, the precise and dynamic regulation of the SLC7A11 stability by the KCTD10–USP18 axis under the conditions of cystine deficiency vs. sufficiency is pathologically relevant and biologically significant.

Inhibition of the cystine/GSH/GPX4 pathway induces ferroptosis and suppresses tumor growth with SLC7A11 as a hub of this axis (6). By modulating the stability of SLC7A11, we reported here that both KCTD10 and USP18 regulate ferroptosis, either positively or negatively, in a SLC7A11-dependent manner. Although KCTD10 was shown to be involved in the growth of different cancer cell lines (52–54), its role in regulation of cell death, particularly ferroptosis, is completely unknown. Here, we showed that KCTD10 knockdown increased the viability and clonogenic survival of breast cancer cells by stabilizing SLC7A11 to confer resistance to ferroptosis, which was abrogated by either SLC7A11 knockdown or treatment with a ferroptosis inducer. Thus, KCTD10 suppressed the survival of breast cancer cells by promoting ubiquitylation and degradation of SLC7A11 to induce ferroptosis.

USP18 is a multifunctional protein and is implicated in a variety of pathological conditions including cancer (55). One recent study showed that USP18 inhibition induces pyroptotic cell death (56). Although USP18 was shown to regulate lipid metabolism (57), no study was reported to demonstrate its role in ferroptosis. We showed here that USP18 knockdown reduced cell viability by destabilizing SLC7A11 to induce ferroptosis, which is blocked by either ectopic expression of SLC7A11 or treatment with a ferroptosis inhibitor. Thus, in contrast to KCTD10, USP18 promotes the survival of breast cancer cells by stabilizing SLC7A11 to suppress ferroptosis. Our correlation study using the human breast cancer tissues also revealed a negative association between KCTD10 and SLC7A11, and a positive association between USP10 and SLC7A11 at both mRNA and protein levels, indicating a pathological relevance of our finding. Finally, we showed that the combination of MLN4924 with Erastin or IKE exhibits a better activity against the growth and survival of breast cancer cells in both in vitro cell-culture setting and in vivo xenograft tumor models, providing a proof-of-concept evidence and sound strategy for effective anticancer therapy by MLN4924 in combination of SLC7A11 inhibitors.

In summary, our study fits the following working model: MLN4924 inactivates CRL3 to cause accumulation of SLC7A11, which is a new substrate of CRL3^KCTD10^ for ubiquitylation and degradation, and of USP18 for deubiquitylation and stabilization. Upon cystine starvation, the levels of KCTD10 or USP18 are decreased or increased, respectively, to cause accumulation of SLC7A11, leading to enhanced cystine uptake/consumption and resistance to ferroptosis (Fig. 7*L*). The combination of MLN4924 and SLC7A11 inhibitor IKE effectively blocks tumor growth, revealing a sound therapeutic strategy.

## Materials and Methods

The use of human breast cancer tissues in the study was approved by the ethical committee of Sir Run Run Shaw Hospital, Zhejiang University School of Medicine, China. Informed consent was obtained from all of the subjects involved as previously described (15). Immunoblots and IP, protein half-life measurement, in vivo ubiquitylation assay, metabolic profiling analysis, and cell viability and clonogenic survival assay were performed as previously described (14, 15). Additional materials and methods are available in *SI Appendix*, *SI Materials and Methods*.

## Supplementary Material

Appendix 01 (PDF)

## Data Availability

Fragments per kilobase million (FPKM) normalized expression profile data from The Cancer Genome Atlas (TCGA) was downloaded from UCSC Xena data hub (https://xena.ucsc.edu/) (58) using R (version 4.2.0) and R packages. All other study data are included in the article and/or *SI Appendix*.

## References

[1] Y. Fuchs, H. Steller, Programmed cell death in animal development and disease. Cell **147**, 742–758 (2011). 22078876 10.1016/j.cell.2011.10.033 PMC4511103

[2] E. Koren, Y. Fuchs, Modes of regulated cell death in cancer. Cancer Discov. **11**, 245–265 (2021). 33462123 10.1158/2159-8290.CD-20-0789

[3] D. Hanahan, R. A. Weinberg, Hallmarks of cancer: The next generation. Cell **144**, 646–674 (2011). 21376230 10.1016/j.cell.2011.02.013

[4] S. J. Dixon , Ferroptosis: An iron-dependent form of nonapoptotic cell death. Cell **149**, 1060–1072 (2012). 22632970 10.1016/j.cell.2012.03.042 PMC3367386

[5] B. R. Stockwell, Ferroptosis turns 10: Emerging mechanisms, physiological functions, and therapeutic applications. Cell **185**, 2401–2421 (2022). 35803244 10.1016/j.cell.2022.06.003 PMC9273022

[6] X. Jiang, B. R. Stockwell, M. Conrad, Ferroptosis: Mechanisms, biology and role in disease. Nat. Rev. Mol. Cell Biol. **22**, 266–282 (2021). 33495651 10.1038/s41580-020-00324-8 PMC8142022

[7] E. Nakamura , 4F2 (CD98) heavy chain is associated covalently with an amino acid transporter and controls intracellular trafficking and membrane topology of 4F2 heterodimer. J. Biol. Chem. **274**, 3009–3016 (1999). 9915839 10.1074/jbc.274.5.3009

[8] P. Koppula, L. Zhuang, B. Gan, Cystine transporter SLC7A11/xCT in cancer: Ferroptosis, nutrient dependency, and cancer therapy. Protein Cell **12**, 599–620 (2021). 33000412 10.1007/s13238-020-00789-5 PMC8310547

[9] W. S. Yang , Regulation of ferroptotic cancer cell death by GPX4. Cell **156**, 317–331 (2014). 24439385 10.1016/j.cell.2013.12.010 PMC4076414

[10] S. Zhang, Q. Yu, Z. Li, Y. Zhao, Y. Sun, Protein neddylation and its role in health and diseases. Signal Transduct. Target Ther. **9**, 85 (2024). 38575611 10.1038/s41392-024-01800-9 PMC10995212

[11] T. A. Soucy, L. R. Dick, P. G. Smith, M. A. Milhollen, J. E. Brownell, The NEDD8 conjugation pathway and its relevance in cancer biology and therapy. Genes Cancer **1**, 708–716 (2010). 21779466 10.1177/1947601910382898 PMC3092238

[12] T. A. Soucy , An inhibitor of NEDD8-activating enzyme as a new approach to treat cancer. Nature **458**, 732–736 (2009). 19360080 10.1038/nature07884

[13] Q. Zhou, Y. Zheng, Y. Sun, Neddylation regulation of mitochondrial structure and functions. Cell Biosci. **11**, 55 (2021). 33731189 10.1186/s13578-021-00569-6 PMC7968265

[14] Q. Zhou , Inhibiting neddylation modification alters mitochondrial morphology and reprograms energy metabolism in cancer cells. JCI Insight **4**, e121582 (2019). 30668548 10.1172/jci.insight.121582 PMC6478410

[15] Q. Zhou , Neddylation inhibition induces glutamine uptake and metabolism by targeting CRL3(SPOP) E3 ligase in cancer cells. Nat. Commun. **13**, 3034 (2022). 35641493 10.1038/s41467-022-30559-2 PMC9156729

[16] L. A. Timmerman , Glutamine sensitivity analysis identifies the xCT antiporter as a common triple-negative breast tumor therapeutic target. Cancer Cell **24**, 450–465 (2013). 24094812 10.1016/j.ccr.2013.08.020 PMC3931310

[17] A. Muir , Environmental cystine drives glutamine anaplerosis and sensitizes cancer cells to glutaminase inhibition. ELife **6**, e27713 (2017). 28826492 10.7554/eLife.27713 PMC5589418

[18] L. Yang, S. Venneti, D. Nagrath, Glutaminolysis: A hallmark of cancer metabolism. Annu. Rev. Biomed. Eng. **19**, 163–194 (2017). 28301735 10.1146/annurev-bioeng-071516-044546

[19] C. Yoshimura , TAS4464, a highly potent and selective inhibitor of NEDD8-activating enzyme, suppresses neddylation and shows antitumor activity in diverse cancer models. Mol. Cancer Ther. **18**, 1205–1216 (2019). 31092565 10.1158/1535-7163.MCT-18-0644

[20] H. Zhou , Selective inhibition of cullin 3 neddylation through covalent targeting DCN1 protects mice from acetaminophen-induced liver toxicity. Nat. Commun. **12**, 2621 (2021). 33976147 10.1038/s41467-021-22924-4 PMC8113459

[21] J. Cheng , Functional analysis of Cullin 3 E3 ligases in tumorigenesis. Biochim. Biophys. Acta Rev. Cancer **1869**, 11–28 (2018). 29128526 10.1016/j.bbcan.2017.11.001 PMC7076836

[22] T. Liu, L. Jiang, O. Tavana, W. Gu, The deubiquitylase OTUB1 mediates ferroptosis via stabilization of SLC7A11. Cancer Res. **79**, 1913–1924 (2019). 30709928 10.1158/0008-5472.CAN-18-3037 PMC6467774

[23] M. Maekawa, S. Higashiyama, KCTD10 biology: An adaptor for the ubiquitin E3 complex meets multiple substrates: Emerging divergent roles of the cullin-3/KCTD10 E3 ubiquitin ligase complex in various cell lines. Bioessays **42**, e1900256 (2020). 32484264 10.1002/bies.201900256

[24] T. Nagai, S. Mukoyama, H. Kagiwada, N. Goshima, K. Mizuno, Cullin-3-KCTD10-mediated CEP97 degradation promotes primary cilium formation. J. Cell Sci. **131**, jcs219527 (2018). 30404837 10.1242/jcs.219527

[25] E. Gasol, M. Jimenez-Vidal, J. Chillaron, A. Zorzano, M. Palacin, Membrane topology of system xc- light subunit reveals a re-entrant loop with substrate-restricted accessibility. J. Biol. Chem. **279**, 31228–31236 (2004). 15151999 10.1074/jbc.M402428200

[26] M. J. Clague, S. Urbe, D. Komander, Breaking the chains: Deubiquitylating enzyme specificity begets function. Nat. Rev. Mol. Cell Biol. **20**, 338–352 (2019). 30733604 10.1038/s41580-019-0099-1

[27] M. Zhang , USP18 recruits USP20 to promote innate antiviral response through deubiquitinating STING/MITA. Cell Res. **26**, 1302–1319 (2016). 27801882 10.1038/cr.2016.125 PMC5143414

[28] C. S. Shin , The glutamate/cystine xCT antiporter antagonizes glutamine metabolism and reduces nutrient flexibility. Nat. Commun. **8**, 15074 (2017). 28429737 10.1038/ncomms15074 PMC5413954

[29] H. Sato , Transcriptional control of cystine/glutamate transporter gene by amino acid deprivation. Biochem. Biophys. Res. Commun. **325**, 109–116 (2004). 15522208 10.1016/j.bbrc.2004.10.009

[30] Q. Chen , SOCS2-enhanced ubiquitination of SLC7A11 promotes ferroptosis and radiosensitization in hepatocellular carcinoma. Cell Death Differ. **30**, 137–151 (2023). 35995846 10.1038/s41418-022-01051-7 PMC9883449

[31] Y. Zhu , TRIM26 induces ferroptosis to inhibit hepatic stellate cell activation and mitigate liver fibrosis through mediating SLC7A11 ubiquitination. Front. Cell Dev. Biol. **9**, 644901 (2021). 33869196 10.3389/fcell.2021.644901 PMC8044755

[32] S. Huang , The deubiquitinase ZRANB1 is an E3 ubiquitin ligase for SLC7A11 and regulates ferroptotic resistance. J. Cell Biol. **222**, e202212072 (2023). 37831441 10.1083/jcb.202212072 PMC10570852

[33] Z. Wang , The DUBA-SLC7A11-c-Myc axis is critical for stemness and ferroptosis. Oncogene **42**, 2688–2700 (2023), 10.1038/s41388-023-02744-0. 37537342

[34] S. J. Dixon , Pharmacological inhibition of cystine-glutamate exchange induces endoplasmic reticulum stress and ferroptosis. Elife **3**, e02523 (2014). 24844246 10.7554/eLife.02523 PMC4054777

[35] M. A. Badgley , Cysteine depletion induces pancreatic tumor ferroptosis in mice. Science **368**, 85–89 (2020). 32241947 10.1126/science.aaw9872 PMC7681911

[36] R. Skouta , Ferrostatins inhibit oxidative lipid damage and cell death in diverse disease models. J. Am. Chem. Soc. **136**, 4551–4556 (2014). 24592866 10.1021/ja411006a PMC3985476

[37] Y. Zhang , Imidazole ketone erastin induces ferroptosis and slows tumor growth in a mouse lymphoma model. Cell Chem. Biol. **26**, 623–633.e9 (2019). 30799221 10.1016/j.chembiol.2019.01.008 PMC6525071

[38] L. Jiang , Ferroptosis as a p53-mediated activity during tumour suppression. Nature **520**, 57–62 (2015). 25799988 10.1038/nature14344 PMC4455927

[39] E. Habib, K. Linher-Melville, H. X. Lin, G. Singh, Expression of xCT and activity of system xc(-) are regulated by NRF2 in human breast cancer cells in response to oxidative stress. Redox Biol. **5**, 33–42 (2015). 25827424 10.1016/j.redox.2015.03.003 PMC4392061

[40] D. Chen , ATF4 promotes angiogenesis and neuronal cell death and confers ferroptosis in a xCT-dependent manner. Oncogene **36**, 5593–5608 (2017). 28553953 10.1038/onc.2017.146 PMC5633655

[41] L. Ma , The m(6)A reader YTHDC2 inhibits lung adenocarcinoma tumorigenesis by suppressing SLC7A11-dependent antioxidant function. Redox Biol. **38**, 101801 (2021). 33232910 10.1016/j.redox.2020.101801 PMC7691619

[42] B. Zhang , LncRNA HEPFAL accelerates ferroptosis in hepatocellular carcinoma by regulating SLC7A11 ubiquitination. Cell Death Dis. **13**, 734 (2022). 36008384 10.1038/s41419-022-05173-1 PMC9411508

[43] Y. Zhang , BAP1 links metabolic regulation of ferroptosis to tumour suppression. Nat. Cell Biol. **20**, 1181–1192 (2018). 30202049 10.1038/s41556-018-0178-0 PMC6170713

[44] Y. Gu , mTORC2 regulates amino acid metabolism in cancer by phosphorylation of the cystine-glutamate antiporter xCT. Mol. Cell **67**, 128–138.e7 (2017). 28648777 10.1016/j.molcel.2017.05.030 PMC5521991

[45] X. Wu , Regulation of TRIF-mediated innate immune response by K27-linked polyubiquitination and deubiquitination. Nat. Commun. **10**, 4115 (2019). 31511519 10.1038/s41467-019-12145-1 PMC6739404

[46] M. Maekawa , Cullin-3/KCTD10 complex is essential for K27-polyubiquitination of EIF3D in human hepatocellular carcinoma HepG2 cells. Biochem. Biophys. Res. Commun. **516**, 1116–1122 (2019). 31280863 10.1016/j.bbrc.2019.07.010

[47] I. Kovacevic , The Cullin-3-Rbx1-KCTD10 complex controls endothelial barrier function via K63 ubiquitination of RhoB. J. Cell Biol. **217**, 1015–1032 (2018). 29358211 10.1083/jcb.201606055 PMC5839774

[48] K. N. Swatek, D. Komander, Ubiquitin modifications. Cell Res. **26**, 399–422 (2016). 27012465 10.1038/cr.2016.39 PMC4822133

[49] A. Kobayashi , Oxidative stress sensor Keap1 functions as an adaptor for Cul3-based E3 ligase to regulate proteasomal degradation of Nrf2. Mol. Cell Biol. **24**, 7130–7139 (2004). 15282312 10.1128/MCB.24.16.7130-7139.2004 PMC479737

[50] I. Lassot , ATF4 degradation relies on a phosphorylation-dependent interaction with the SCF(betaTrCP) ubiquitin ligase. Mol. Cell Biol. **21**, 2192–2202 (2001). 11238952 10.1128/MCB.21.6.2192-2202.2001 PMC86853

[51] B. T. Finicle, V. Jayashankar, A. L. Edinger, Nutrient scavenging in cancer. Nat. Rev. Cancer **18**, 619–633 (2018). 30097614 10.1038/s41568-018-0048-x

[52] T. Ma , KCTD10 functions as a tumor suppressor in hepatocellular carcinoma by triggering the Notch signaling pathway. Am. J. Transl. Res. **15**, 125–137 (2023). 36777839 PMC9908486

[53] Y. Wang , KCTD10 interacts with proliferating cell nuclear antigen and its down-regulation could inhibit cell proliferation. J. Cell Biochem. **106**, 409–413 (2009). 19125419 10.1002/jcb.22026

[54] D. Kubota , Gene expression network analysis of ETV1 reveals KCTD10 as a novel prognostic biomarker in gastrointestinal stromal tumor. PLoS One **8**, e73896 (2013). 23977394 10.1371/journal.pone.0073896 PMC3747077

[55] P. Dziamalek-Macioszczyk, J. Harazna, T. Stompor, Versatility of USP18 in physiology and pathophysiology. Acta Biochim. Pol. **66**, 389–392 (2019). 31747454 10.18388/abp.2019_2844

[56] K. I. Arimoto , Expansion of interferon inducible gene pool via USP18 inhibition promotes cancer cell pyroptosis. Nat. Commun. **14**, 251 (2023). 36646704 10.1038/s41467-022-35348-5 PMC9842760

[57] X. Liu , The ubiquitin-specific peptidase USP18 promotes lipolysis, fatty acid oxidation, and lung cancer growth. Mol. Cancer Res. **19**, 667–677 (2021). 33380466 10.1158/1541-7786.MCR-20-0579 PMC8026529

[58] M. J. Goldman , Visualizing and interpreting cancer genomics data via the Xena platform. Nat. Biotechnol. **38**, 675–678 (2020). 32444850 10.1038/s41587-020-0546-8 PMC7386072

