# Supplementary material for: The CRL3KCTD10 ubiquitin ligase–USP18 axis coordinately regulates cystine uptake and ferroptosis by modulating SLC7A11
Source: Proc Natl Acad Sci U S A. 2024 Jul 3;121(28):e2320655121. doi: 10.1073/pnas.2320655121 (PMC11252818; doi:10.1073/pnas.2320655121)
Supplement: Supplementary file 1 — Appendix 01 (PDF) [file pnas.2320655121.sapp.pdf]

Supplemental Information for

The CRL3^{KCTD10} ubiquitin ligase-USP18 axis coordinately regulates cystine uptake and ferroptosis by modulating SLC7A11

Qiyin Zhou, Hongfei Yu, Yongxia Chen, Jiayi Ren, Yan Lu, and Yi Sun

Correspondence to:

Yi Sun, Email: yisun@zju.edu.cn

This PDF file includes:

SI Materials and Methods

Figures S1 to S8

Legends for Figures S1 to S8

Supplemental Materials and Methods

Cell culture and transfection

Human breast cancer cell lines MDA-MB-231, BT549, SK-BR-3, MCF7, colon cancer cell lines HCT116, RKO, and embryonic kidney cell line HEK293 were obtained from American Type Culture Collection. Human breast cancer cell line SUM159, BT474, and T47D were kind gifts from Dr. Chenfang Dong, Zhejiang University. BT549, BT474, and T47D cells were cultured in RPMI 1640 medium (Gibco), whereas MDA-MB-231, SK-BR-3, MCF7, SUM159, and HEK293 were cultured in DMEM medium (Gibco), and HCT116 was cultured in McCoy's 5A medium (Gibco), supplemented with 10% fetal bovine serum (Gibco).

Cells were transfected with various plasmids using Lipofectamine 3000 Transfection Reagent or with si-RNA oligonucleotides by Lipofectamine RNAiMAX Transfection Reagent, according to the manufacturer's instructions, respectively.

Antibodies and reagents

The following antibodies were used: β-actin (Sigma-Aldrich, A5441, dilution: 1:10000); CUL-1 (Santa Cruz, sc-11384, dilution: 1:1000); CUL-2 (Abcam, ab166917, dilution: 1:1000); CUL-3 (Cell Signaling Technology, 2759S, dilution: 1:1000); CUL-4A (Cell Signaling Technology, 2699S, dilution: 1:1000); CUL-4B (Proteintech, 12916-1-AP, dilution: 1:1000); CUL-5 (Santa Cruz, sc-13014, dilution: 1:1000); FLAG, clone M2 (Sigma-Aldrich, F1804-500UG, dilution: 1:2000); DUBA (Proteintech, 21002-1-AP, dilution: 1:1000); FLAG M2 affinity gel (Sigma-Aldrich, A2220-5ML); 3×FLAG peptide (MCE, HY-P0319A); HA (Sigma, H6908, dilution: 1:2000); Anti-HA High Affinity (3F10) (Roche, 11867423001); KCTD10 (Proteintech, 27279-1-AP, dilution: 1:1000); KCTD10 (Sigma-Aldrich, HPA014273, dilution: 1:1000); KLHL13 (Proteintech, 15612-1-AP, dilution: 1:1000); KLHL25 (Proteintech, 25066-1-AP, dilution: 1:1000); NAEβ (Abcam, ab124728, dilution: 1:1000); USP18 (Cell Signaling Technology, D4E7, dilution: 1:1000); USP18 (ABclonal, A16739, dilution: 1:1000); Recombinant Anti-Sodium Potassium ATPase antibody (Abcam, ab76020); SLC3A2 (Proteintech, 15193-1-AP, dilution: 1:10000); SLC7A11 (Cell Signaling Technology, D2M7A, dilution: 1:1000); SLC7A11 (Novus, NB300-318,

dilution: 1:1000); SOCS2 (Signalway Antiboday, 49110, dilution: 1:1000); TRIM26 (Proteintech, 27013-1-AP, dilution: 1:1000); α-tubulin (Sigma, Clone AA13, T8203, dilution: 1:10000); OTUB1 (ABclonal, A11656, dilution: 1:1000); Peroxidase AffiniPure Goat Anti-Rabbit IgG (H+L) (Jackson, 11-035-144, dilution: 1:4000); Peroxidase AffiniPure Goat Anti-Mouse IgG (H+L) (Jackson, 115-035-146, dilution: 1:4000); Peroxidase AffiniPure Goat Anti-Rat IgG (H+L) (Jackson, 112-035-143, dilution: 1:4000); Ubiquitin (Santa Cruz, P4D1, sc-8017, dilution: 1:1000); ZRANB1 (Abcam, ab103417, dilution: 1:1000) was gift from Dr. Peijing Zhang (Huazhong University of Science and Technology, China).

Reagents were obtained from the following suppliers: Chlorhexidine (CHX) (Sigma, C7698); Ferrostatin-1 (APExBIO, A4371); MG132 (MedChem Express, HY-13259); Erastin (Selleck, S7242); HPBCD (Topscience, T27563); IKE (Selleck, S8877); Lipofectamine RNAiMAX Transfection Reagent (Invitrogen, 13778-150); Lipofectamine 3000 Transfection Reagent (Invitrogen, L3000-015); Ni-NTA Agarose (Qiagen, 30210); MLN4924 (ApexBio, B1036); Puromycin (VWR Life Science, 0336C120); Recombinant Human Ubiquitin Activating Enzyme (UBE1) (R&D Systems, E-305-025); Recombinant Human Ubiquitin Conjugating Enzyme His6-Cdc34/UbcH3 (R&D Systems, E2-610-100) and UbcH5a (R&D Systems, E2-616-100); Recombinant Human Ubiquitin Protein (U-100H-10M); RSL3 (Topscience, T3646).

Plasmids, si-RNAs, and shRNAs

A variety of USP plasmids were gifts from Dr. Lingqiang Zhang (Beijing Institute of Lifeomics, China) and USP18 truncated plasmids were gifts from Dr. Bo Zhong (Wuhan University, China). The si-RNAs were synthesized by Genepharma (Shanghai, China). The sequence of KCTD10 in HBLV-mCherry-KCTD10-PURO lentivirus (Hanbio Tech, Shanghai, China) is as follows: GCCAAGTACTACCTAGTCCAA. Guide RNA of USP18 was cloned into a lentiCRISPR v2 vector (Addgene, 52961) and the sequence of it is as follows: AGGGCACGTTGCACTTCTGC. The sequences of si-RNAs are as follows: CUL-1#1: 5’- GCUCUACACUCAUGUUUAU-3’; CUL-1#2: 5’- GAACCCAGUU

ACUGAAUAU -3'; CUL-2#1: 5'- GCCCUUACGUCAGUUGUAAAUUACA-3'; CUL-2#2: 5'- GA GCUAGCAUUGGAUAUGUGG-3'; CUL-3#1: 5'- GCACAUGAAGACUAUAGU A-3'; CUL-3#2: 5'- GAGUGUAUGAGUUC CUAUU-3'; CUL-4A#1: 5'- GAAGC UGGUCAUCAAGAAC-3'; CUL-4A#2: 5'- GAACUUCCGAGACAGACCU-3'; CUL-4B#1: 5'- AAGCCUAAAUUACC AGAAA-3'; CUL-4B#2: 5'- CACCGUCUCUAGC UUUGCUAA-3'; CUL-5#1: 5'- GUCUCACUUCCUACUGAACUG-3'; CUL-5#2: 5'- CUGGAGGACUUGAU ACCGGAA-3'; NAEβ#1, 5'-GCUUCUCUGCAAAUGAAAU-3; NAEβ#2, 5'-GCUACCAGAACACUGTAUU-3'; USP18#1: 5'- GCCAGAUCCUU CCAAUGAA-3'; USP18#2: 5'- GCGAGAGUCUUGUGAUGCU-3'; SLC7A11#1: 5'- CCAGAUAUGCAUCGUCCUUTT-3'; SLC7A11#2: 5'- CCAGGUGGUUU AGAAUAAUTT -3'; KCTD10#1: 5'- GCUGAAGUCUGUUGUACCU-3'; KCTD10#2: 5'- GUAACAACAAAUACUCAUA-3'.

The *in vitro* ubiquitylation assay

For *in vitro* ubiquitylation assay, HEK293 cells were transfected with HA-tagged SLC7A11 or FLAG-tagged KCTD10, individually. Approximately 48 h later, the transfected cells were harvested. After RIPA buffer Lysis, HA-tagged SLC7A11 was purified with anti-HA beads, and KCTD10 was purified with anti-FLAG beads and eluted with 3×FLAG peptide. Then purified SLC7A11 and KCTD10 were incubated with 2 μg/μl Ubiquitin, 2 ng/μl UBE1 (E1), 10 ng/μl His6-Cdc34 (E2), and 10 ng/μl UbcH5a (E2) in a ubiquitin reaction buffer (50 mM Tris pH 7.4, 5 mM $MgCl_2$, 2 mM NaF, 0.1mg/ml BSA, 2 mM ATP, 0.6 mM DTT) for 90 min under constant vortexing at 37°C and polyubiquitinated SLC7A11 was analyzed by SDS-PAGE.

For *in vitro* deubiquitylation assay, HEK293 cells were transfected with HA-SLC7A11 and His-Ub for 48 hr and treated with 10 μM MG132 for 6 h before harvest. Then cells were lysed in RIPA buffer, and ubiquitinated SLC7A11 was purified by anti-HA beads. Flag-USP18 was purified from transfected HEK293 cells with anti-Flag beads and eluted with 3×FLAG peptide. Purified ubiquitinated SLC7A11 was then incubated with purified USP18 in deubiquitination buffer (50 mM

Tris-HCl, 5 mM $MgCl_2$, 5% glycerol, pH 7.5) for 5 hr at 37 °C and analyzed by SDS-PAGE.

Quantitative real-time PCR assay

Briefly, total cellular RNA was isolated using a TRIzol Reagent (Ambion, 15596018), and RNase-free DNase (Takara, 2270A) was added to eliminate DNA contamination. The first strand cDNA was synthesized using a PrimeScript™ RT reagent Kit (Perfect Real Time) (Takara, RR037Q). About 100 ng cDNA from each sample was used to analyze gene expression with SYBR *Premix EX Taq* (Takara, RR420A). The quantitative real-time PCR primers specific for each gene are listed below (5'-3'): *β-ACT*-F: TCACCCACACTGTGCCCATCTAC, *β-ACT*-R: GGAACCGCTCATTGCCAATG; *SLC7A11*-F: GTCCGCAAGCACAC TCCTCTAC, *SLC7A11*-R, CGAATAGAGGGAAAGGGCAAC; *KCTD10*-F, AGC CGTGAAGTTGCTCTACA, *KCTD10*-R, GGAAACTCCACCTTGGTCTGTT; *USP18*-F, CCTGGAAGTGAAGTCGTGCT, *USP18*-R, CTTCTCCTCTGCTCGT CAGC.

Immunostaining and confocal microscopy

Cells grown on coverslips were fixed with 4% paraformaldehyde solution for 10 min. The coverslips were treated with 0.5% Triton-X100 to permeabilize and then incubated in PBS/FBS (PBS, pH7.4, containing 5% FBS) to block nonspecific sites of antibody absorption. Then, the cells were incubated with appropriate primary and secondary antibodies as indicated in the figure legends. Images were captured on a Leica TCS SP8 DIVE laser scanning confocal microscope.

Measurement of cystine consumption

Cystine uptake/comsumption was measured with Cystine Uptake Assay Kit (Dojindo, UP05). Cystine uptake/consumption was calculated by deducting the measured cystine concentration in the medium from the original cystine. All values were normalized according to cell number.

The *in vivo* tumorigenesis assay

All animal studies were approved by and conducted in accordance with the guidelines established by the committee on Use and Care of Animals at the Zhejiang University.

These mice were housed in the specific pathogen-free (SPF) environment at a constant temperature (25℃) and a relatively constant humidity with ad libitum access to water and food (40-60%), with 12 h dark/light cycle.

MDA-MB-231 cells (4×10^6) were injected subcutaneously. When the tumor size reached approximately 100 mm^3, the mice were randomized. MLN4924 (30 mg/kg) or vehicle control was given to mice by subcutaneous injection, 5 days a week, for two weeks; IKE (40 mg/kg) was given to mice via intraperitoneal injection, once every other day, for two weeks. Mice in the drug control group received 10% 2-hydroxypropyl-β-cyclodextrin (HPBCD) as the vehicle control. Tumor size and body weight were measured at the indicated time points and average tumor volumes were calculated according to the formula (length × width × width)/2. At the end of experiment, tumors were harvested and weighed.

In a separate experiment, MDA-MB-231 cells (4×10^6) expressing indicated shRNAs or sgRNAs were injected subcutaneously. When the tumor size reached approximately 100 mm^3, the mice were randomized. IKE (40 mg/kg) was given to mice via intraperitoneal injection, once every other day, for two weeks. Mice in the drug control group received 10% 2-hydroxypropyl-β-cyclodextrin (HPBCD) as the vehicle control. Tumor size and body weight were measured at the indicated time points and average tumor volumes were calculated according to the formula (length × width × width)/2. At the end of experiment, tumors were harvested and weighed.

Immunohistochemical staining and image analysis

Human breast tumor tissue arrays were purchased from Xi'an Taibs Pharmarceutical Technology Co., Ltd. For immune-histochemical staining, the sections were deparaffinized in xylene and rehydrated through graded ethanol. Antigen retrieval was performed for 20min at 95 °C with 0.1% sodium citrate buffer (pH 6.0). Following quenching of endogenous peroxidase activity with 3%

H_2O_2·dH2O and blocking of non-specific binding with 1% bovine serum albumin buffer, sections were incubated overnight at 4 °C with an anti-SLC7A11 (Novus, NB300-318, 1:400 dilution), anti-KCTD10 (Proteintech, 27279-1-AP, 1:100 dilution), or anti-USP18 (ABclonal, A16739, dilution: 1:1000) antibody. Following several washes, the sections were treated with HRP conjugated secondary antibody for 30min at room temperature, and stained with 0.05% 3, 3-diaminobenzidine tetrahydrochloride (DAB). Images were taken from a scanner (KFBIO, KF-FL-020).

Bioinformatic analysis

Fragments per kilobase million (FPKM) normalized expression profile data from The Cancer Genome Atlas (TCGA) was downloaded from UCSC Xena data hub (https://xena.ucsc.edu/). All statistical analyses were performed using R (version 4.2.0) and R packages.

Statistical analysis

All statistical analysis was performed using the GraphPad Prism version 8.0.2.263 and SPSS 16.0. All statistical comparisons were evaluated by the Student's t-test or one-way or two-way analysis of variance (ANOVA). Two-sided Student's t-test was used to analyze the differences between normal samples and tumor samples. In addition, all the graphs or statistical significance for bioinformatic analysis in this study were produced by R (version 4.2.0). Among all the data sets, p values less than 0.05 were considered significant.

Supplementary Figures

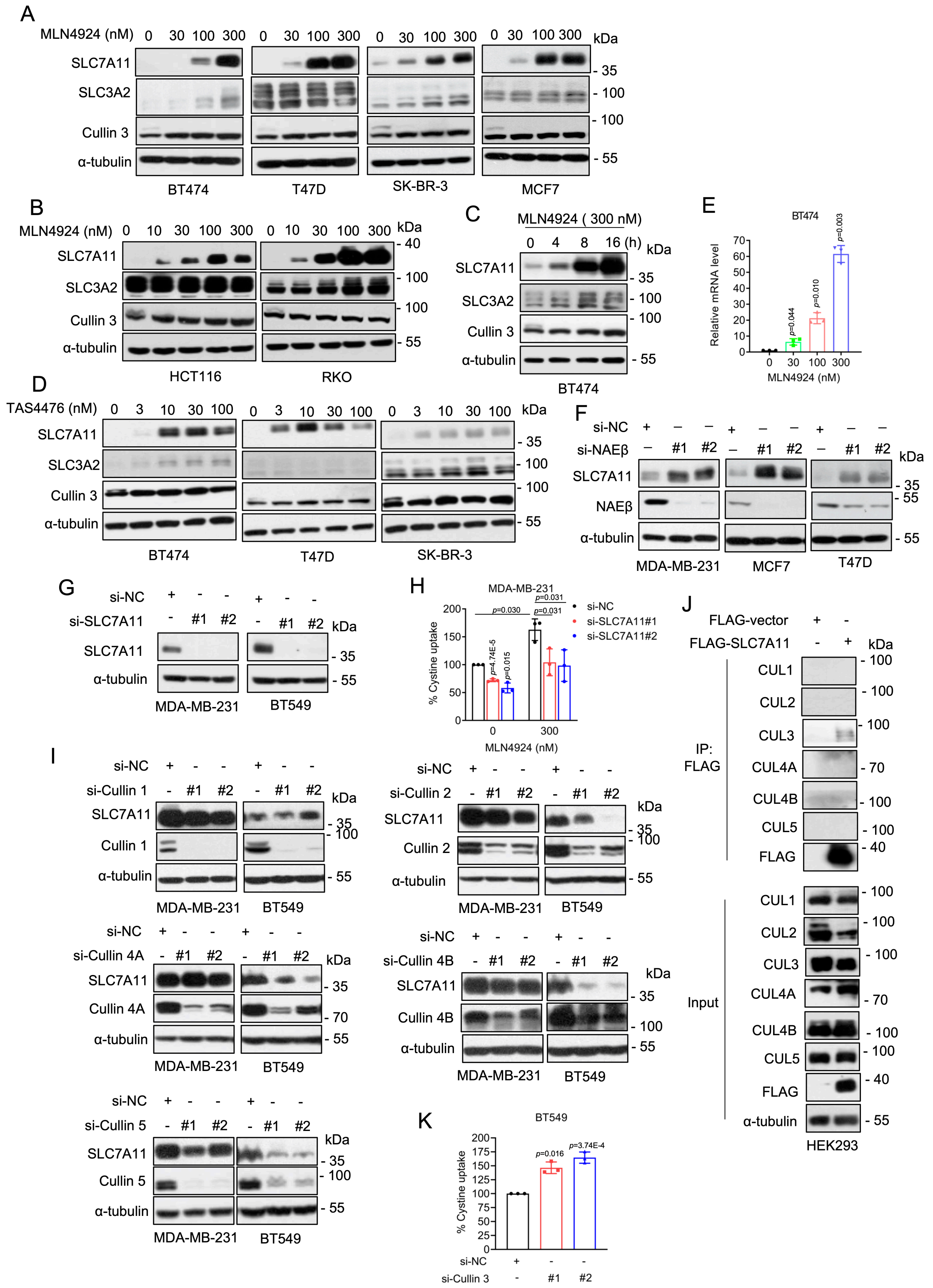

***SI Appendix*, Fig. S1. MLN4924 inactivates CRL3 E3 ligase increases cystine uptake through causing SLC7A11 accumulation.**

A BT474, T47D, SK-BR-3, and MCF7 cells were treated with indicated concentrations of MLN4924 for 24h and analyzed by immunoblotting.

B HCT116 and RKO cells were treated with indicated concentrations of MLN4924 for 24h and analyzed by immunoblotting.

C BT474 cells were treated with 300 nM MLN4924 for various time and analyzed by immunoblotting.

D BT474, T47D, and SK-BR-3 cells were treated with indicated concentrations of TAS4464 for 24h and analyzed by immunoblotting.

E BT474 cells were treated with MLN4924 for 24 h, followed by qRT-PCR analysis (mean ± SD, n= 3).

F MDA-MB-231, MCF7, and T47D were transfected with siRNA targeting NAEβ or scramble control siRNA for 48h, followed by immunoblotting.

G MDA-MB-231 and BT549 cells were transfected with siRNA targeting SLC7A11 or scramble control siRNA for 48h, followed by immunoblotting.

H MDA-MB-231 cells were transfected with indicated siRNAs (si-NC or si-SLC7A11s) against SLC7A11 for 24h, then treated with 300 nM MLN4934, followed by cystine uptake detection after 24h (mean ± SD, n=3).

I MDA-MB-231 and BT549 cells were transfected with indicated siRNAs targeting Cullin 1, Cullin 2, Cullin 4A, Cullin 4B, and Cullin 5, respectively, for 48h, and then subjected to immunoblotting.

J HEK293 cells were transfected with FLAG-SLC7A11 plasmid, immunoprecipitated with FLAG-agarose beads and analyzed by immunoblotting.

K BT549 cells were transfected with indicated siRNAs (si-NC or si-Cullin 3s) againt Cullin 3 for 48h, followed by cystine uptake detection (mean ± SD, n=3).

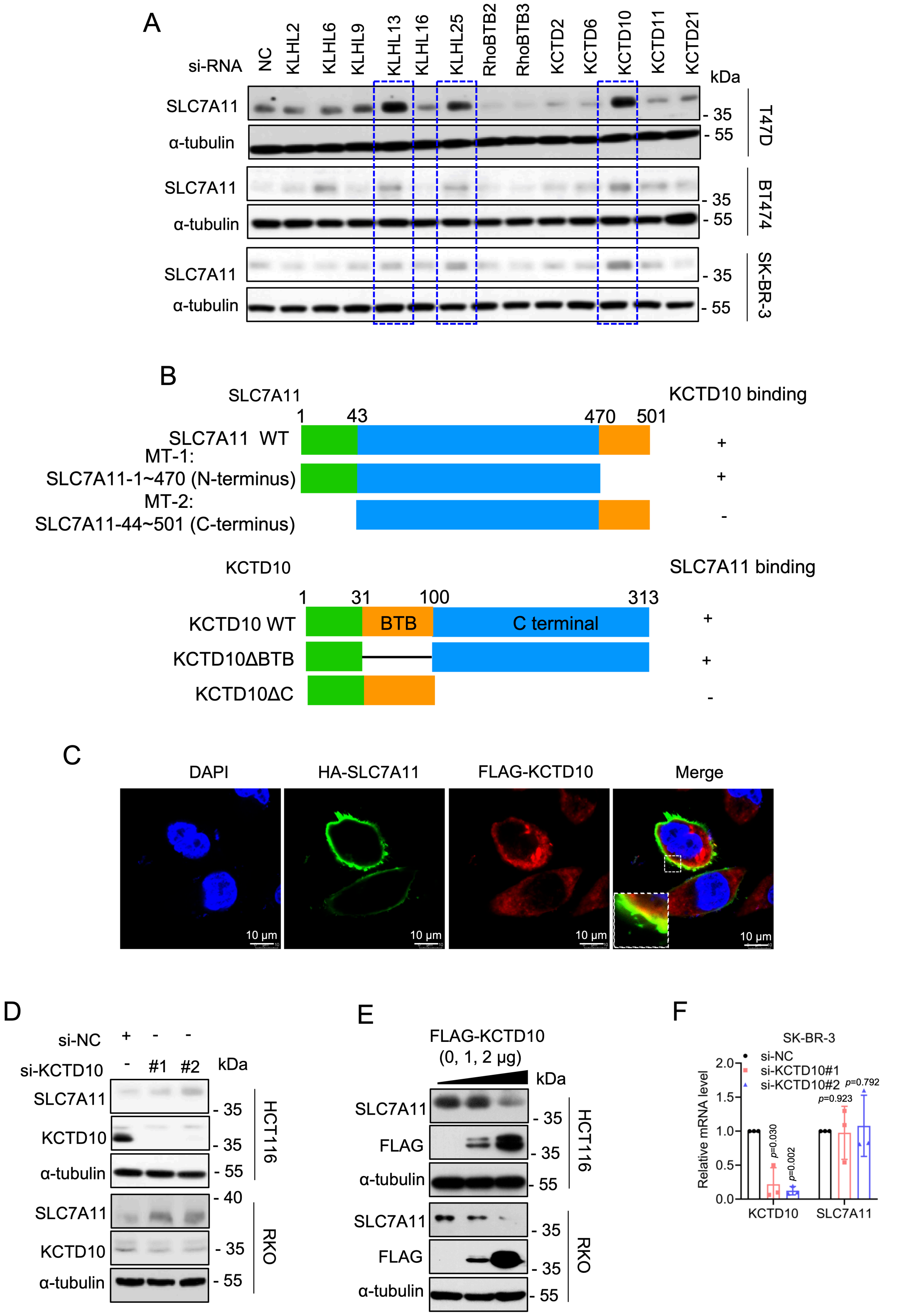

A
si-RNA
NC
KLHL2
KLHL6
KLHL9
KLHL13
KLHL16
KLHL25
RhoBTB2
RhoBTB3
KCTD2
KCTD6
KCTD10
KCTD11
KCTD21
kDa
SLC7A11
α-tubulin
- 35
- 55
T47D
BT474
SK-BR-3
B
SLC7A11
KCTD10 binding
1
43
470
501
SLC7A11 WT
MT-1:
SLC7A11-1~470 (N-terminus)
MT-2:
SLC7A11-44~501 (C-terminus)
+
+
-
KCTD10
SLC7A11 binding
1
31
100
313
KCTD10 WT
BTB
C terminal
KCTD10ΔBTB
KCTD10ΔC
+
+
-
C
DAPI
HA-SLC7A11
FLAG-KCTD10
Merge
10 μm
D
si-NC
si-KCTD10
+ - -
- #1 #2
kDa
SLC7A11
KCTD10
α-tubulin
- 35
- 35
- 55
- 40
- 35
- 55
HCT116
RKO
E
FLAG-KCTD10
(0, 1, 2 μg)
kDa
SLC7A11
FLAG
α-tubulin
- 35
- 35
- 55
HCT116
RKO
F
SK-BR-3
si-NC
si-KCTD10#1
si-KCTD10#2
Relative mRNA level
2.0
1.5
1.0
0.5
0.0
p=0.030
p=0.002
p=0.923
p=0.792
KCTD10
SLC7A11

***SI Appendix*, Fig. S2. KCTD10 negative regulates SLC7A11 level.**

A T47D, BT474, and SK-BR-3 cells were transfected with siRNA targeting various CRL3 adaptor proteins or scramble control siRNA for 48h, followed by immunoblotting.

B Summary of the binding domains on SLC7A11 and KCTD10.

C BT549 cells were transfected with HA-SLC7A11 and FLAG-KCTD10 plasmids, and their colocalization was analyzed through immunofluorescence.

D HCT116 and RKO cells were transfected with siRNA targeting KCTD10 or scramble control siRNA for 48h, followed by immunoblotting.

E HCT116 and RKO cells were transfected with increasing amounts plasmids encoding KCTD10 for 48h, followed by immunoblotting.

F SK-BR-3 cells were transfected with siRNA targeting KCTD10 or scramble control siRNA for 48h, followed by qRT-PCR analysis (mean ± SD, n= 3).

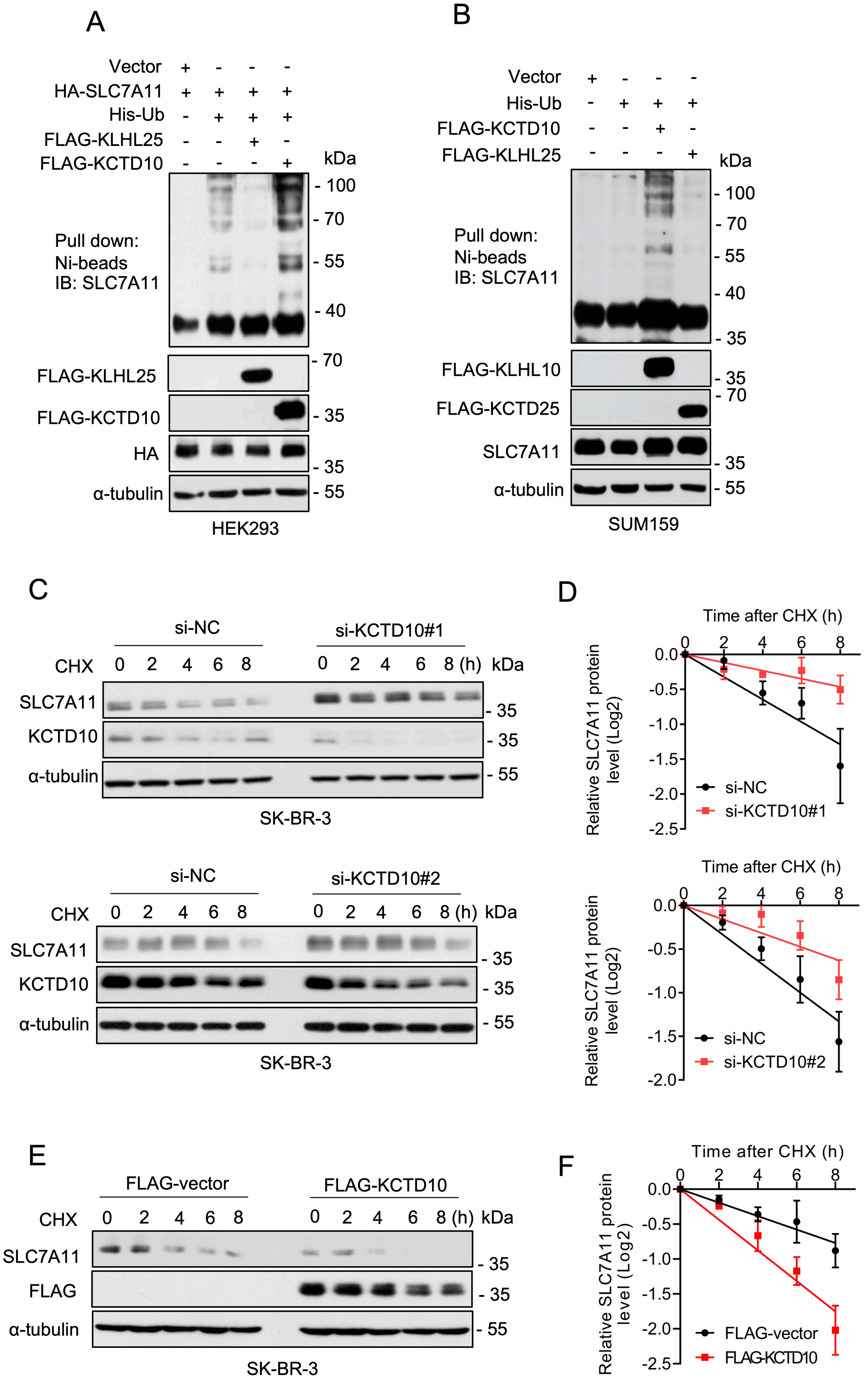
A
Vector
HA-SLC7A11
His-Ub
FLAG-KLHL25
FLAG-KCTD10
kDa
Pull down:
Ni-beads
IB: SLC7A11
FLAG-KLHL25
FLAG-KCTD10
HA
α-tubulin
HEK293
B
Vector
His-Ub
FLAG-KCTD10
FLAG-KLHL25
Pull down:
Ni-beads
IB: SLC7A11
FLAG-KLHL10
FLAG-KCTD25
SLC7A11
α-tubulin
SUM159
C
si-NC
si-KCTD10#1
CHX
0 2 4 6 8 (h)
SLC7A11
KCTD10
α-tubulin
SK-BR-3
si-NC
si-KCTD10#2
SK-BR-3
D
Time after CHX (h)
Relative SLC7A11 protein level (Log2)
si-NC
si-KCTD10#1
si-KCTD10#2
E
FLAG-vector
FLAG-KCTD10
FLAG
SK-BR-3
F
FLAG-vector
FLAG-KCTD10

SI Appendix**, Fig. S3. SLC7A11 stability is negative regulated by KCTD10.**

A HEK293 cells were transfected with indicated ubiquitin plasmids, lysed under denaturing conditions, followed by Ni-beads pulldown and immunoblotting for SLC7A11.

B SUM159 cells were transfected with indicated plasmids, followed by Ni-beads pulldown and immunoblotting for SLC7A11.

C&D SK-BR-3 cells were transfected with siRNA targeting KCTD10 (si-KCTD10#1 or KCTD10#2) or scramble siRNA for 48h, then incubated with cycloheximide (CHX). Cells were harvested at indicated periods of time for immunoblotting (C). The band density of SLC7A11 was quantified using Image J software and normalized to α-tubulin to draw a decay curve (mean ± SD, n=3), respectively (D).

E&F BT549 cells were transfected with plasmid encoding FLAG-KCTD10, along with the vector control for 48h, then incubated with cycloheximide (CHX). Cells were harvested at indicated periods of time for immunoblotting (E). The band density of SLC7A11 was quantified using Image J software and normalized to α-tubulin to draw a decay curve (mean ± SD, n=3), respectively (F).

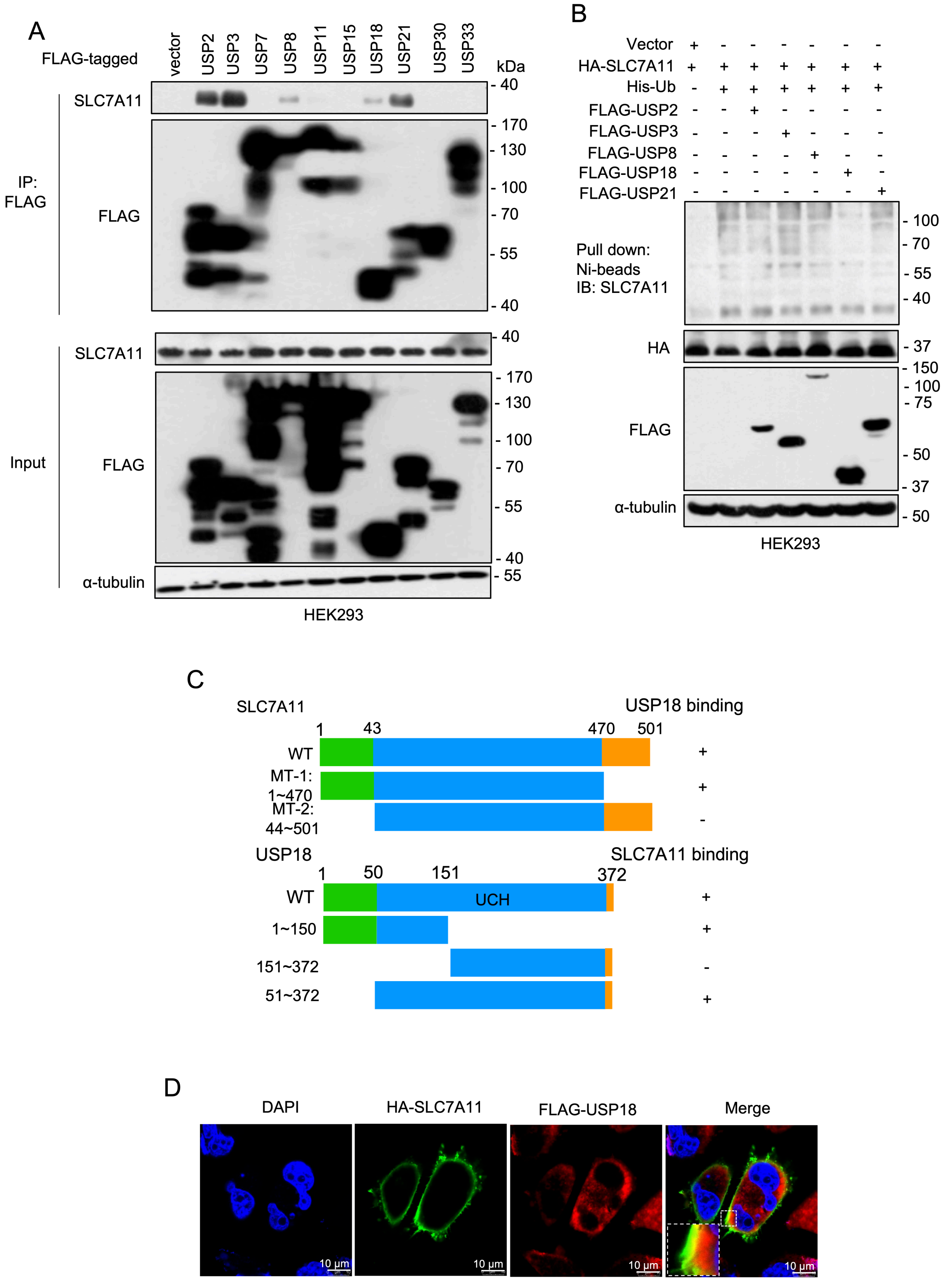
A
FLAG-tagged
vector
USP2
USP3
USP7
USP8
USP11
USP15
USP18
USP21
USP30
USP33
kDa
IP: FLAG
SLC7A11
FLAG
Input
SLC7A11
FLAG
α-tubulin
HEK293
B
Vector
HA-SLC7A11
His-Ub
FLAG-USP2
FLAG-USP3
FLAG-USP8
FLAG-USP18
FLAG-USP21
Pull down: Ni-beads
IB: SLC7A11
HA
FLAG
α-tubulin
HEK293
C
SLC7A11
USP18 binding
WT
MT-1: 1~470
MT-2: 44~501
USP18
SLC7A11 binding
WT
UCH
1~150
151~372
51~372
D
DAPI
HA-SLC7A11
FLAG-USP18
Merge
10 μm

***SI Appendix*, Fig. S4. USP18 interacts with SLC7A11.**

A HEK293 cells were transfected with indicated FLAG-tagged plasmids, immunoprecipitated with FLAG-agarose beads and analyzed by immunoblotting.

B HEK293 cells were transfected with indicated plasmids, lysed under denaturing conditions, followed by Ni-beads pulldown and immunoblotting for SLC7A11.

C Summary of the binding domains on SLC7A11 and USP18.

D BT549 cells were transfected with HA-SLC7A11 and FLAG-USP18 plasmids, and their colocalization was analyzed through immunofluorescence.

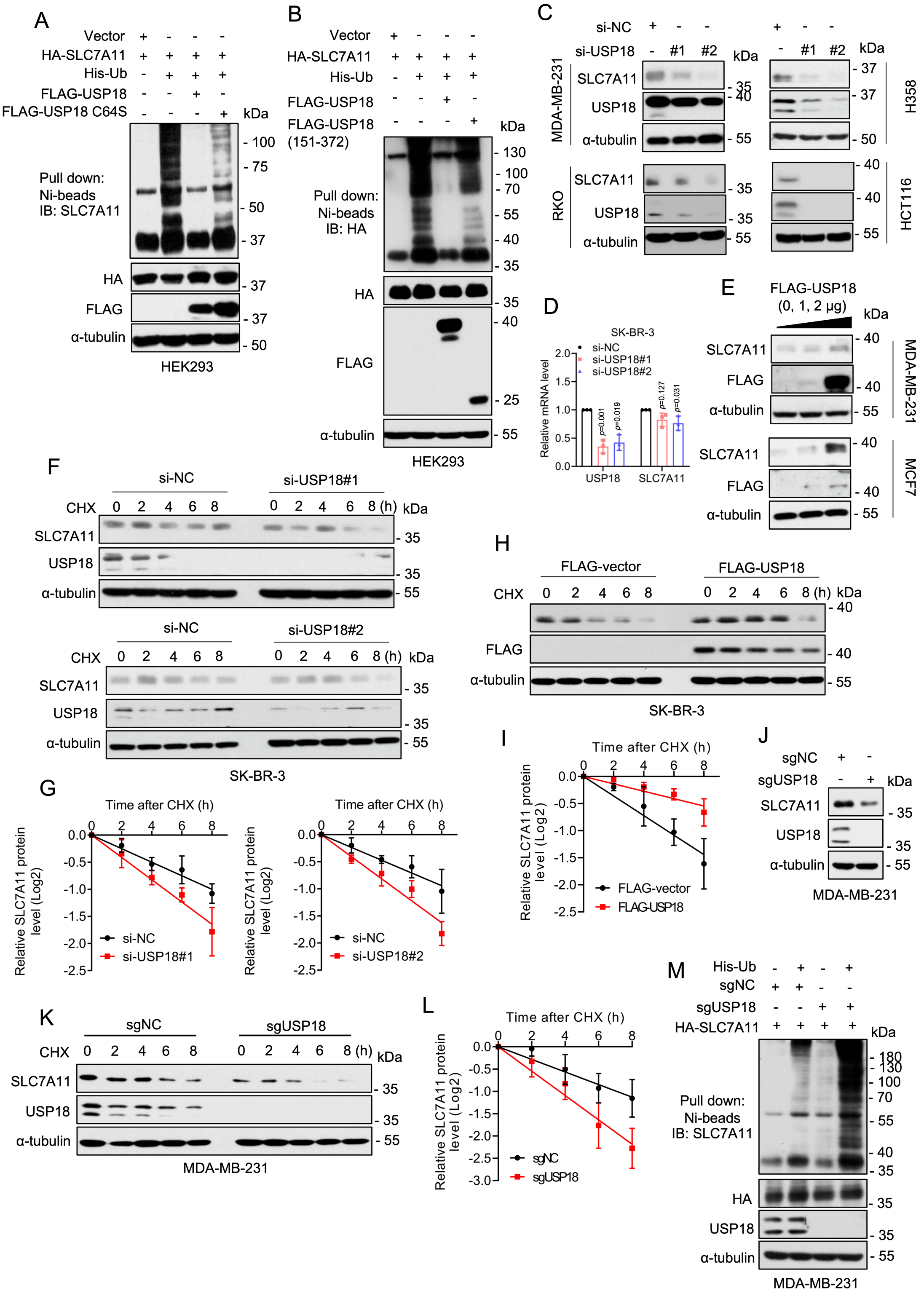
A
Vector
HA-SLC7A11
His-Ub
FLAG-USP18
FLAG-USP18 C64S
Pull down: Ni-beads IB: SLC7A11
HA
FLAG
α-tubulin
HEK293
B
FLAG-USP18 (151-372)
Pull down: Ni-beads IB: HA
C
si-NC
si-USP18
MDA-MB-231
RKO
H358
HCT116
USP18
D
SK-BR-3
Relative mRNA level
p=0.001
p=0.019
p=0.127
p=0.031
E
FLAG-USP18 (0, 1, 2 μg)
MCF7
F
CHX
si-USP18#1
si-USP18#2
G
Time after CHX (h)
Relative SLC7A11 protein level (Log2)
H
FLAG-vector
I
J
sgNC
sgUSP18
K
L
M
kDa

***SI Appendix*, Fig. S5. USP18 positively regulates SLC7A11 stability.**

A&B HEK293 cells were transfected with indicated plasmids, lysed under denatured conditions, Ni-bead pulldown, and immunoblotting for SLC7A11 (A) or HA (B).

C MDA-MB-31, H358, RHO, and HCT116 cells were transfected with siRNA targeting USP18 (si-USP18#1 or USP18#2) or scramble siRNA for 48h, and analyzed by immunoblotting.

D SK-BR-3 cells were transfected with si-NC control or two independent si-USP18s targeting USP18 for 48h, followed by qRT-PCR analysis (mean ± SD, n =3).

E MDA-MB-231 and MCF7 cells were transfected with increasing amounts of plasmids encoding USP18 for 48h, and analyzed by immunoblotting.

F&G SK-BR-3 cells were transfected with siRNA targeting USP18 (si-USP18#1 or USP18#2) or scramble siRNA for 48h, then incubated with cycloheximide (CHX). Cells were harvested at indicated periods of time for immunoblotting (F). The band density of SLC7A11 was quantified using Image J software and normalized to α-tubulin to draw a decay curve (mean ± SD, n=3), respectively (G).

H&I SK-BR-3 cells were transfected with FLAG-vector or FLAG-USP18 for 48h, then incubated with cycloheximide (CHX). Cells were harvested at indicated periods of time for immunoblotting (H). The band density of SLC7A11 was quantified using Image J software and normalized to α-tubulin to draw a decay curve (mean ± SD, n=3), respectively (I).

J MDA-MB-231 cells without or with USP18 deletion were analyzed by immunoblotting.

K&L MDA-MB-231 cells without or with USP18 deletion incubated with cycloheximide (CHX), and harvested at indicated periods of time for immunoblotting (K). The band density of SLC7A11 was quantified using Image J software and normalized to α-tubulin to draw a decay curve (mean ± SD, n=3), respectively (L).

M MDA-MB-231 cells without or with USP18 deletion were transfected with indicated plasmids, lysed under denaturing conditions, followed by Ni-beads pulldown and immunoblotting for SLC7A11.

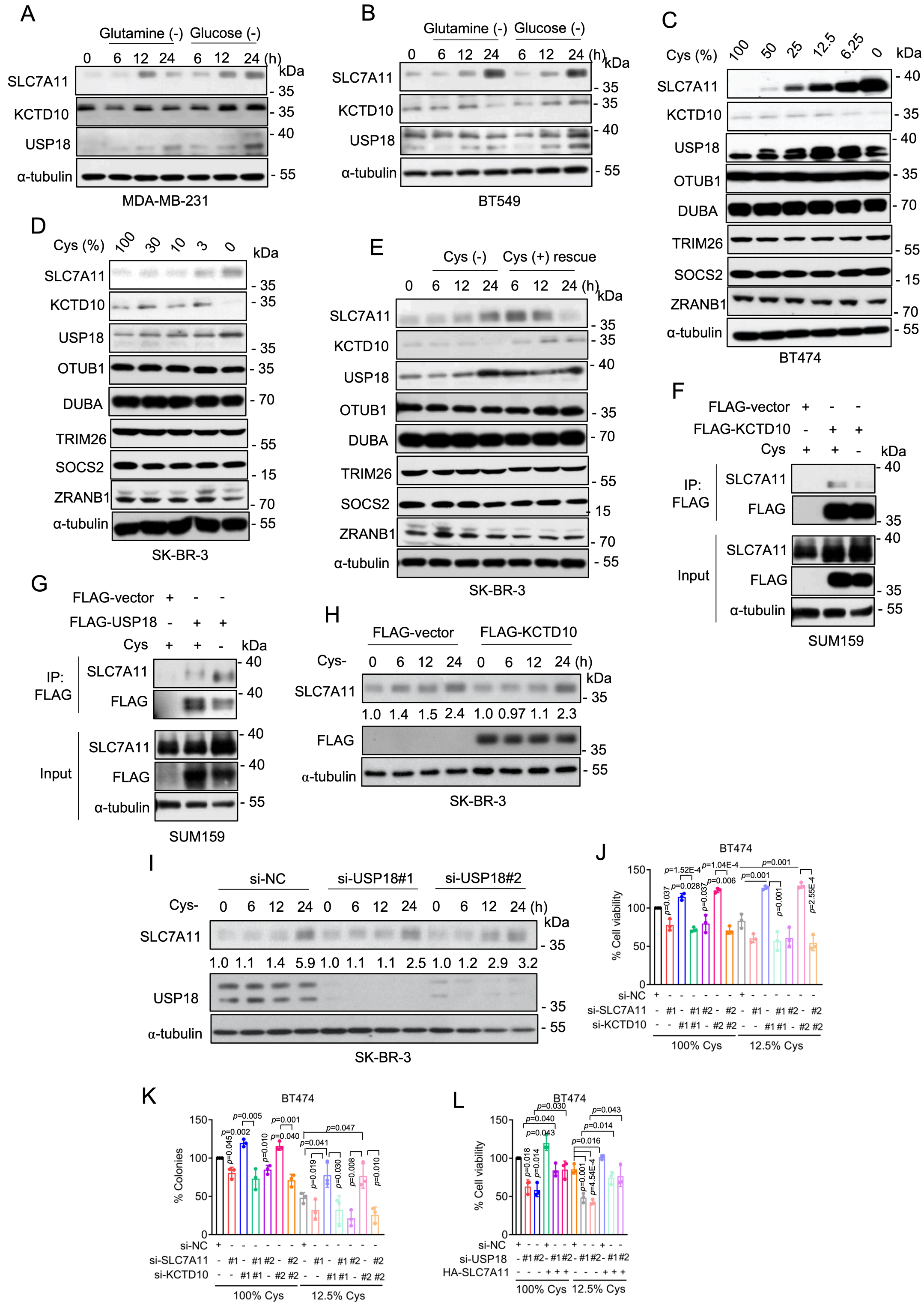

A
Glutamine (-) Glucose (-)
0 6 12 24 6 12 24 (h)
kDa
SLC7A11
KCTD10
USP18
α-tubulin
- 35
- 35
- 40
- 55
MDA-MB-231
B
Glutamine (-) Glucose (-)
0 6 12 24 6 12 24 (h)
kDa
SLC7A11
KCTD10
USP18
α-tubulin
- 35
- 35
- 40
- 55
BT549
C
Cys (%) 100 50 25 12.5 6.25 0
kDa
SLC7A11
KCTD10
USP18
OTUB1
DUBA
TRIM26
SOCS2
ZRANB1
α-tubulin
- 40
- 35
- 40
- 35
- 70
- 55
- 15
- 70
- 55
BT474
D
Cys (%) 100 30 10 3 0
kDa
SLC7A11
KCTD10
USP18
OTUB1
DUBA
TRIM26
SOCS2
ZRANB1
α-tubulin
- 35
- 35
- 35
- 35
- 70
- 55
- 15
- 70
- 55
SK-BR-3
E
Cys (-) Cys (+) rescue
0 6 12 24 6 12 24 (h)
kDa
SLC7A11
KCTD10
USP18
OTUB1
DUBA
TRIM26
SOCS2
ZRANB1
α-tubulin
- 35
- 35
- 40
- 35
- 70
- 55
- 15
- 70
- 55
SK-BR-3
F
FLAG-vector + - -
FLAG-KCTD10 - + +
Cys + + -
kDa
IP: FLAG
SLC7A11
FLAG
Input
SLC7A11
FLAG
α-tubulin
- 40
- 35
- 40
- 35
- 55
SUM159
G
FLAG-vector + - -
FLAG-USP18 - + +
Cys + + -
kDa
IP: FLAG
SLC7A11
FLAG
Input
SLC7A11
FLAG
α-tubulin
- 40
- 40
- 40
- 40
- 55
SUM159
H
FLAG-vector FLAG-KCTD10
Cys- 0 6 12 24 0 6 12 24 (h)
kDa
SLC7A11
1.0 1.4 1.5 2.4 1.0 0.97 1.1 2.3
FLAG
α-tubulin
- 35
- 35
- 55
SK-BR-3
I
si-NC si-USP18#1 si-USP18#2
Cys- 0 6 12 24 0 6 12 24 0 6 12 24 (h)
kDa
SLC7A11
1.0 1.1 1.4 5.9 1.0 1.1 1.1 2.5 1.0 1.2 2.9 3.2
USP18
α-tubulin
- 35
- 35
- 55
SK-BR-3
J
BT474
% Cell viability
si-NC
si-SLC7A11
si-KCTD10
100% Cys
12.5% Cys
K
BT474
% Colonies
si-NC
si-SLC7A11
si-KCTD10
100% Cys
12.5% Cys
L
BT474
% Cell viability
si-NC
si-USP18
HA-SLC7A11
100% Cys
12.5% Cys

***SI Appendix*, Fig. S6. Cystine deprivation regulates KCTD10- and USP18-controled ubiquitylation of SLC7A11.**

A&B MDA-MB-231 (A) and BT549 (B) cells were cultured with glucose- and glutamine-free medium for indicated time, then analyzed by immunoblotting.

C&D BT474 (C) and SK-BR-3 (D) cells were cultured with indicated percentage of cystine for 24h for immunoblotting.

E SK-BR-3 cells were cultured with cystine-free medium at different time points, followed by cystine addition for different time points and analyzed by immunoblotting.

F SUM159 cells were transfected with FLAG-KCTD10 plasmid, cultured in cystine-containing or cystine-free media, followed by immunoprecipitattion with FLAG-agarose beads and analyzed by immunoblotting.

G SUM159 cells were transfected with FLAG-USP18 plasmid, cultured in cystine-containing or cystine-free media, followed by immunoprecipitattion with FLAG-agarose beads and analyzed by immunoblotting.

H SK-BR-3 cells were transfected with FLAG-KCTD10 plasmid, along with the vector control for 48h, then cultured with cystine-free medium for different time points, followed by immunoblotting.

I SK-BR-3 cells were transfected with si-NC control or two independent si-USP18s targeting USP18 for 48h, then cultured with cystine-free medium for different time points, followed by immunoblotting.

J&K BT474 cells were transfected with indicated siRNAs and cultured with medium containing 100% and 12.5% cystine, then cell viability (J) and clonogenic survival were measured (mean ± SD, n =3) (K).

L BT474 cells were transfected with indicated siRNAs and plasmids, cultured with medium containing 100% and 12.5% cystine for 48h, then cell viability was measured (mean ± SD, n =3).

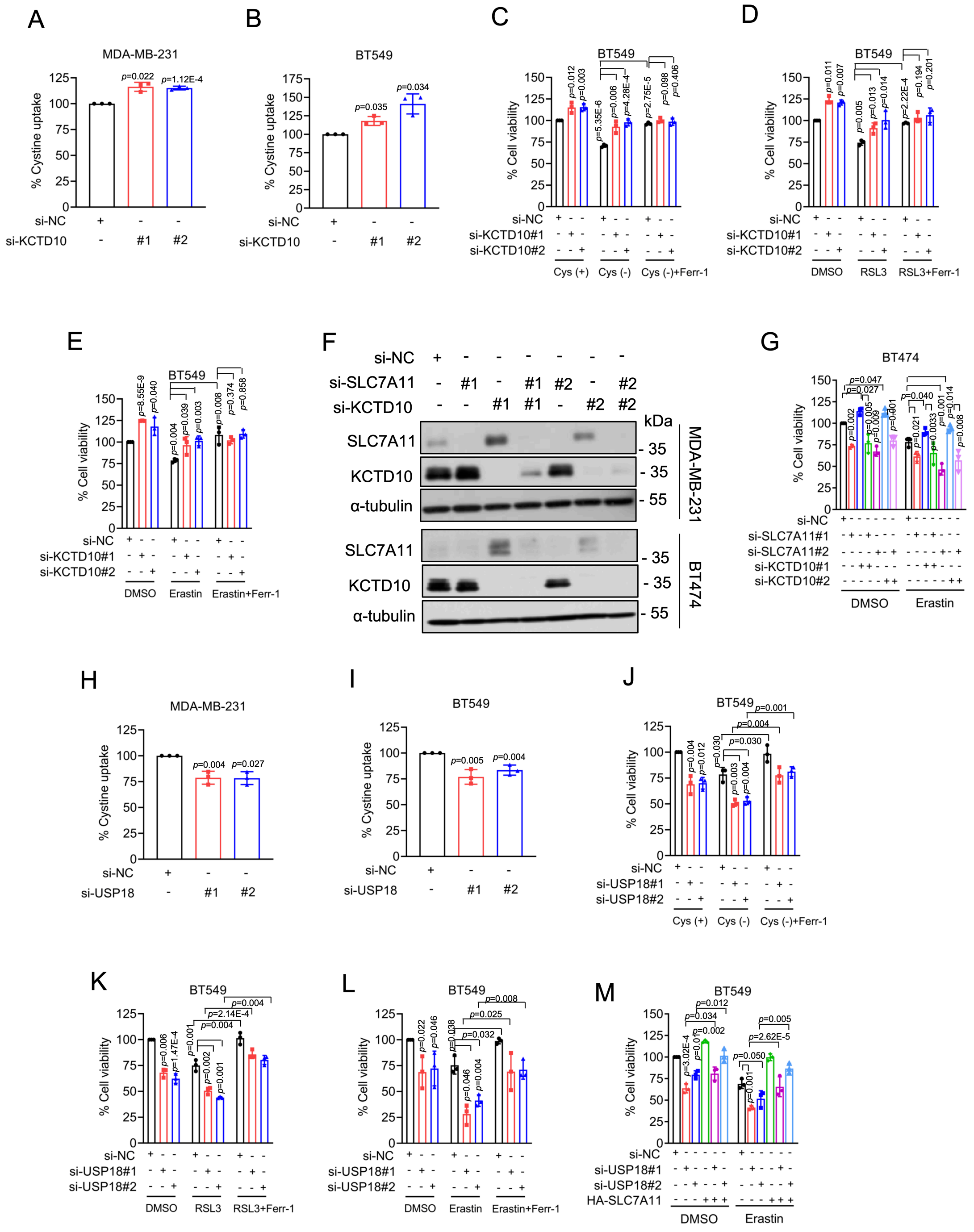

***SI Appendix*, Fig. S7. KCTD10 and USP18 regulate ferroptosis through SLC7A11.**

A&B MDA-MB-231 (A) and BT549 (B) cells were transfected with indicated siRNAs (si-NC or si-KCTD10s), followed by cystine uptake detection (mean ± SD, n=3).

C-E BT549 cells were transfected with indicated siRNAs (si-NC or si-KCTD10s), followed by cystine deprivation (C), RSL3 (D) and Erastin (E) treatment or together with Ferrostatin-1 for 24h as indicated, then cell viability was measured (mean ± SD, n =3).

F MDA-MB-231 and BT474 cells were transfected with indicated siRNAs and subjected to immunoblotting.

G BT474 cells transfected with indicated siRNAs (si-NC or si-KCTD10) for 24h, then treated without and with Erastin for 48h, then cell viability was measured (mean ± SD, n =3).

H&I MDA-MB-231 (H) and BT549 (I) cells were transfected with indicated siRNAs (si-NC or si-USP18s), followed by cystine uptake detection (mean ± SD, n=3).

J-L BT549 cells were transfected with indicated siRNAs (si-NC or si-USP18s), followed by cystine deprivation (J), RSL3 (K) and Erastin (L) treatment or together with Ferrostatin-1 for 24h as indicated, then cell viability was measured (mean ± SD, n =3).

M BT549 cells transfected with indicated siRNAs (si-NC or si-USP18s) and plasmids (HA-SLC7A11) for 24h, then treated without and with Erastin for 48h, then cell viability was measured (mean ± SD, n =3).

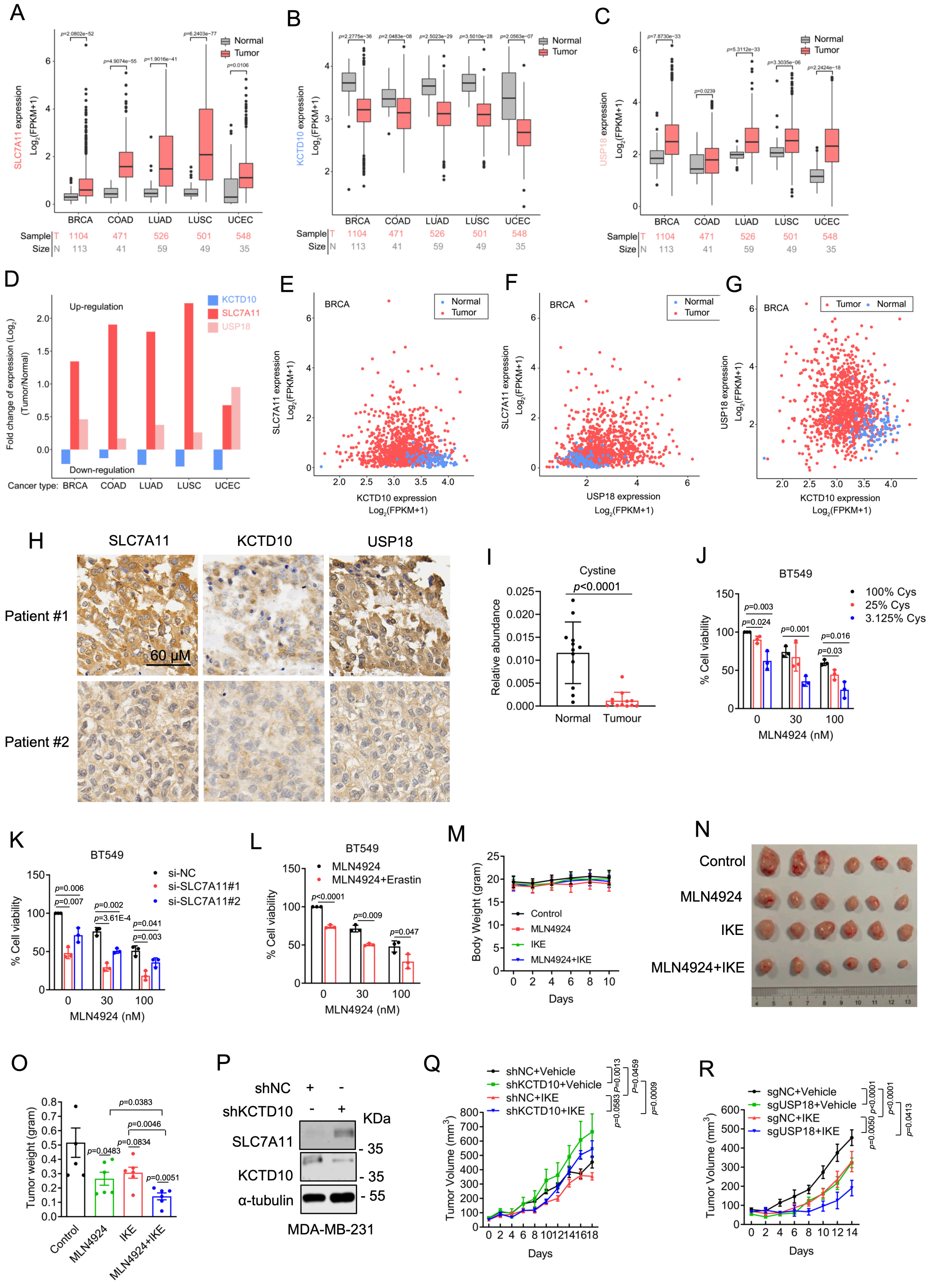

A
B
C
Normal
Tumor
SLC7A11 expression
KCTD10 expression
USP18 expression
Log2(FPKM+1)
BRCA
COAD
LUAD
LUSC
UCEC
Sample Size
T
N
1104
471
526
501
548
113
41
59
49
35
D
Up-regulation
Down-regulation
Fold change of expression (Log2) (Tumor/Normal)
KCTD10
SLC7A11
USP18
Cancer type:
E
F
G
KCTD10 expression
USP18 expression
H
SLC7A11
KCTD10
USP18
Patient #1
Patient #2
60 μM
I
Cystine
p<0.0001
Relative abundance
Normal
Tumour
J
BT549
100% Cys
25% Cys
3.125% Cys
% Cell viability
MLN4924 (nM)
K
si-NC
si-SLC7A11#1
si-SLC7A11#2
L
MLN4924
MLN4924+Erastin
M
Body Weight (gram)
Days
Control
MLN4924
IKE
MLN4924+IKE
N
O
Tumor weight (gram)
P
shNC
shKCTD10
KDa
SLC7A11
KCTD10
α-tubulin
MDA-MB-231
Q
shNC+Vehicle
shKCTD10+Vehicle
shNC+IKE
shKCTD10+IKE
Tumor Volume (mm3)
R
sgNC+Vehicle
sgUSP18+Vehicle
sgNC+IKE
sgUSP18+IKE

***SI Appendix*, Fig. S8. KCTD10 and USP18 correlate with SLC7A11 and co-targeting neddylation and SLC7A11 enhances cancer killing.**

A-D Differential expression of SLC7A11, KCTD10, and USP18 in tumor tissues and adjacent normal tissues. Expression data were obtained from TCGA datasets. BRCA, breast cancer; COAD, colon adenocarcinoma; LUAD, lung adenocarcinoma; LUSC, lung squamous cell carcinoma; UCEC, Uterine Corpus Endometrial Carcinoma.

E-G Expression levels of KCTD10 and SLC7A11 (E), USP18 and SLC7A11 (F), and KCTD10 and USP18 (G) in individual breast cancer patient samples. Red dots, tumor tissues. Blue dots, adjacent normal tissues.

H Representative images of SLC7A11, KCTD10, and USP18 staining in breast tissues (normal vs. tumor).

I Paired human breast normal and tumor tissues were analyzed by targeted metabolomics, and relative abundance of cystine evaluated/compared between total of normal vs. tumor tissues. n=12 with mean ± SD.

J BT549 cells were treated with MLN4924 under various percentage of cystine in culture media for 48 h, and the number of viable cells was quantified by trypan blue exclusion assay (mean ± SD, n= 3).

K BT549 cells were transfected with scramble control siRNA (si-NC) or siRNA targeting SLC7A11 (si-SLC7A11s) and then treated with DMSO control or MLN4924, followed by trypan blue exclusion assay for cell viability after 48 h (mean ± SD, n = 3).

L BT549 cells were treated with different concentrations of MLN4924 without or with 1.25 μM Erastin for 48 h and cell viability was measured by trypan blue exclusion assay (mean ± SD, n = 3).

M-O The *in vivo* growth of MDA-MB-231 xenograft tumors after 14 days of treatment of MLN4924, or IKE, alone or in combination, along with vehicle control and body weight was monitored (M). The tumor mass were photographed (N) and weighted (O) at the end of experiment.

P MDA-MB-231 cells with stable knockdown of KCTD10 along with the shNC

control, followed by immunoblotting.

Q MDA-MB-231 cells with stable knockdown of KCTD10 along with the shNC control were subcutaneously injected to nude mice individually, treated with IKE or vehicle control. The *in vivo* tumor growth was measured and plotted (mean ± SEM, n =6).

R MDA-MB-231 cells with deletion of USP18 along with the sgNC control were subcutaneously injected to nude mice individually, treated with IKE or vehicle control. The *in vivo* tumor growth was measured and plotted (mean ± SEM, n =5).
